# A Machine Learning Approach in Analyzing Bioaccumulation of Heavy Metals in Turbot Tissues

**DOI:** 10.3390/molecules25204696

**Published:** 2020-10-14

**Authors:** Ștefan-Mihai Petrea, Mioara Costache, Dragoș Cristea, Ștefan-Adrian Strungaru, Ira-Adeline Simionov, Alina Mogodan, Lacramioara Oprica, Victor Cristea

**Affiliations:** 1 Department of Foood Science, Food Engineering, Biotechnology and Aquaculture, Faculty of Food Science and Engineering, University “Dunărea de Jos” of Galați, 800008 Galați, Romania; ira.simionov@gmail.com (I.-A.S.); alina.antache@ugal.ro (A.M.); victor.cristea@ugal.ro (V.C.); 2 The Fish Culture Research and Development Station of Nucet, 137335 Dâmbovița-Nucet, Romania; 3 Faculty of Economics and Business, University “Dunărea de Jos” of Galați, 800008 Galați, Romania; dragoscristea@yahoo.com; 4 Institute for Interdisciplinary Research, Science Research Department, “Alexandru Ioan Cuza” University of Iasi, Lascar Catargi Str. 54, 700107 Iasi, Romania; strungaru_stefan@yahoo.com; 5 Multidisciplinary Research Platform (ReForm), University “Dunărea de Jos” of Galați, 800008 Galați, Romania; 6 Department of Biology, Faculty of Biology, Alexandru Ioan Cuza University, 700506 Iasi, Romania; lacramioara.oprica@uaic.ro

**Keywords:** heavy metals, machine learning, prediction models, random forest, turbot

## Abstract

Metals are considered to be one of the most hazardous substances due to their potential for accumulation, magnification, persistence, and wide distribution in water, sediments, and aquatic organisms. Demersal fish species, such as turbot (*Psetta maxima maeotica*), are accepted by the scientific communities as suitable bioindicators of heavy metal pollution in the aquatic environment. The present study uses a machine learning approach, which is based on multiple linear and non-linear models, in order to effectively estimate the concentrations of heavy metals in both turbot muscle and liver tissues. For multiple linear regression (MLR) models, the stepwise method was used, while non-linear models were developed by applying random forest (RF) algorithm. The models were based on data that were provided from scientific literature, attributed to 11 heavy metals (As, Ca, Cd, Cu, Fe, K, Mg, Mn, Na, Ni, Zn) from both muscle and liver tissues of turbot exemplars. Significant MLR models were recorded for Ca, Fe, Mg, and Na in muscle tissue and K, Cu, Zn, and Na in turbot liver tissue. The non-linear tree-based RF prediction models (over 70% prediction accuracy) were identified for As, Cd, Cu, K, Mg, and Zn in muscle tissue and As, Ca, Cd, Mg, and Fe in turbot liver tissue. Both machine learning MLR and non-linear tree-based RF prediction models were identified to be suitable for predicting the heavy metal concentration from both turbot muscle and liver tissues. The models can be used for improving the knowledge and economic efficiency of linked heavy metals food safety and environment pollution studies.

## 1. Introduction

Among all of the pollutants, metals are the most hazardous substances due to their potential for accumulation, magnification, persistence and wide distribution within the water table, sediments, and aquatic organisms [1,2].

In the aquatic environment, fish have been widely utilized as environmental bioindicators of microplastic pollution [3,4,5,6] metal pollution [1,7,8,9,10,11,12,13,14,15,16], sewage sludge pollution [17], suspended solid pollution [18], polychlorinated biphenyl (PCBs) pollution, and polycyclic aromatic hydrocarbon (PAH) pollution [19].

Fish are suitable bioindicators of metal pollution due to their capacity to accumulate higher metal concentrations in their tissues, as compared to the concentrations present in the surrounding water [9,12,16]. By using fish as bioindicators, direct data on the bioavailable fraction of heavy metals can be obtained [20]. Trace metals, such as iron (Fe), zinc (Zn), copper (Cu), cobalt (Co), manganese (Mn), nickel (Ni), chromium (Cr), or selenium (S), are essential elements that are involved in the normal metabolism of fish [2]. Other trace metals, such as lead (Pb) and cadmium (Cd) are non-essential elements and do not have any biological role in the fish’s organism [21]. Both essential and non-essential metals may become toxic above a specific threshold [21]. Furthermore, alkali metals, such as sodium (Na) or potassium (K), and alkaline earth metals, such as calcium (Ca) or magnesium (Mg), influence the bioaccumulation capacity of trace metals in fish [22].

The response of fish to environmental pollution is species specific. It is important that the chosen bioindicator fish species is not migratory and, thus, can accurately indicate pollution levels in a certain study area, have a superior trophic level (carnivorous or piscivorous) [9,10,14,15] and a specific diet [14].

Flatfish, such as the turbot (*Psetta maxima maeotica*, Linnaeus, 1758), are accepted by the scientific communities as good bioindicators of heavy metal pollution in the aquatic environment, due to their association to bottom sediments [21,22,23,24,25,26,27,28,29,30]. The feeding ecology of the turbot is benthivorous, which is, it feeds on organic detritus and small preys inhabiting the sediment superficial layers [14]. In general, toxic metals accumulate more in benthic fish species when compared to pelagic ones [9,11]. Moreover, the turbot does not undertake long and transboundary migrations; therefore it is an important instrument in assessing water pollution [22].

Metal analysis is the most direct procedure for the quantification of these elements in the environment; however, this approach involves high financial costs [20]. The Regional Organization for the Protection of the Marine Environment recommends using collected fish as an environmental monitoring tool in order to minimize the costs related to field sampling studies [12].

Ecotoxicological models can be developed for selected fish species, which have the capacity to accumulate pollutants within their tissues [10]. Several studies have generated prediction models [31,32,33,34,35,36,37,38,39]. For instance, mathematical models (One Compartment Approach) were developed in order to predict internal concentrations of organic chemicals in fish [31]. Other studies developed prediction models of heavy metals in the nematode (*Caenorhabditis elegans*) [32], in the water, and sediment matrix [33,34,35].

Even though the prediction models of heavy metals in fish have been previously approached [36,37,38,39], new prediction models should be developed in order to improve the pre-existing data.

The present study aims to use machine learning to develop multiple linear and non-linear models, in order to effectively estimate the concentrations of heavy metals in both turbot muscle and liver tissues, based on data that were provided from scientific literature, attributed to a maximum number of 11 elements (As, Ca, Cd, Cu, Fe, K, Mg, Mn, Na, Ni, and Zn).

## 2. Results and Discussion

The present research uses multiple linear regression to explain several heavy metal relationships where data allow it. The MLR modelling provides the possibility to determine the relative influence of one or more predictor variables to the criterion value. Using MLR for predicting heavy metals concentration in organic contexts is a common technique.

However, the predicting models involving the available heavy metals parameters were not all linear. Even if the linear models are easier to interpret, there were cases that were described by non-linear relationships among parameters. For these cases, random forest machine learning algorithms were able to identify strong existing data patterns that were formalized as non-linear predictive models that were validated by their high accuracy in predicting previously unseen data samples.

### 2.1. The Correlation Matrix

**Figure 1:**
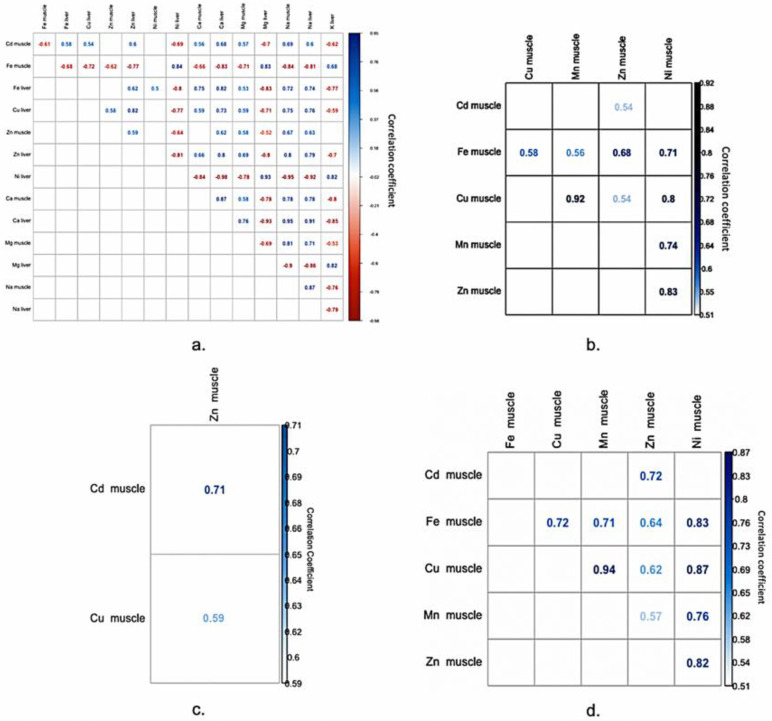
Correlation matrix of heavy metals concentration in turbot tissues ((**a**) correlation matrix for the first group samples; (**b**) correlation matrix for the second group samples; (**c**) correlation matrix for the third group samples; and, (**d**) correlation matrix for the fourth group samples).

In the research at hand, the correlation matrix was used as a tool to summarize the linear relationships existent in our data as well as for identifying strong and relevant relationships that could be further modelled. Therefore, as part of the analytical framework, all of the data related to heavy metals concentration in both turbot muscle and liver tissues were processed using Python NumPy library for obtaining the correlation matrix and Seaborn library to visualize it (Figure 1). Therefore, four correlation matrixes were generated in order to cover the relationships between variables from all 63 samples, grouped in the first four groups mentioned above. The fifth group did not present any significant correlations between the elements; thus, no correlation matrix was generated.

The correlation matrixes display the Pearson correlation coefficients between all of the available variables. It is considered that, if the correlation coefficient between two variables is +0.7, then this reveals a strong positive linear correlation between the two variables; thus, the two parameters are moving in the same direction. Additionally, if the correlation coefficient is negative, the two parameters are moving in opposite directions. Weak positive and negative correlations would be in the range of 0.1 ÷ 0.3/−0.1 ÷ −0.3, moderate positive and negative correlation from 0.3 ÷ 0.5/0.3 ÷ 0.5, and strong positive and negative correlation from 0.5 ÷ 1.0/0.5 ÷ 1.0 [40].

The correlation matrix offers an easy visualization over the linear relations that exists in the dataset for subsequent modelling and it aids in reducing the linear model’s multicollinearity issue by spotting independent predictors that are strongly correlated. This correlation can be a problem, because independent variables are supposed to be independent. If the degree of correlation between variables is high enough, problems can arise when fitting the model and interpreting the results.

#### 2.1.1. Positive Significant Correlations

By analyzing the correlation matrix, significant positive correlations are found between Cd in turbot muscle tissues and Ca, Na, and Zn in liver tissues, respectively, Na in muscle tissues, for the first group (Figure 1a). Positive correlation between Cd and Zn concentration in turbot muscle tissues are observed also for the second, third, and fourth groups (Figure 1b–d)

The Fe concentrations in turbot muscle tissues, in the first group, are positively correlated with K, Mg, and Ni in liver tissues, while Fe concentrations in turbot liver is positively correlated with Ca and Na concentrations in both muscle and liver tissues (Figure 1a). Additionally, correlations between Fe in muscle tissues and Cu, Mn, Zn, and Ni, all from muscle tissues, are recorded for the second and fourth groups (Figure 1b,d).

The Cu concentration in liver tissues, in the first group, is positively correlated with Ca, Na, and Zn in liver, respectively, Na in turbot muscle tissues (Figure 1a). Additionally, when considering the second and fourth groups, Cu in muscle was strongly correlated with Mn and Ni from muscle tissues (Figure 1b,d).

Positive correlations are also observed, in the first group, between Zn concentration in muscle and Ca and Na concentrations in liver, respectively Na in turbot muscle tissues (Figure 1a). Additionally, in the same group, the Zn concentration in the liver is positively correlated with Ca and Na concentration in both liver and muscle tissue, respectively, Mg in turbot muscle tissues (Figure 1a). Additionally, the second and fourth groups registered strong correlations between Zn in turbot muscle and Ni from muscle tissues (Figure 1b,d), while the third group registered a strong correlation between Zn and Cd in muscle tissues (Figure 1c)

The Ni in the liver is positively correlated with K and Mg in turbot liver tissues (Figure 1a), while the Ca concentration in muscle tissue is correlated positively with the Ca in liver tissues, situations valid for the first group (Figure 1a). Furthermore, Ca and Na are positively correlated in both turbot liver and muscle tissues from first group (Figure 1a). The Na concentration in muscle tissue is correlated positively with the Na in liver tissues from the first group (Figure 1a). Other positive correlations are also observed between Mg in muscle and Na in both turbot muscle and liver tissues from the first group (Figure 1a).

#### 2.1.2. Negative Significant Correlations

Negative significant correlations were observed only for the first group (Figure 1a). Thus, the correlation matrix reveals significant negative correlations between Cd in turbot muscle tissues and K, Ni, and Mg in liver tissues, respectively Fe in muscle tissues (Figure 1a). Additionally, the Fe concentration in turbot muscle tissues is negatively correlated with Ca, Cu, Fe, Na, and Zn in liver tissues, respectively, Ca, Mg, Na, and Zn in turbot muscle tissues (Figure 1a). The Fe concentration in the liver is negatively correlated with K, Ni, and Mg concentration in the liver (Figure 1a).

The Cu concentration in liver tissue is negatively correlated with Mg and Ni in liver (Figure 1a). Additionally, Zn is negatively correlated with K, Mg, and Ni in liver tissues (Figure 1a). The Ni concentration in the liver is negatively correlated with Ca and Na in turbot liver tissues, respectively, Ca and Mg in muscle tissues (Figure 1a). The Ca in both analyzed tissues is negatively correlated with K and Mg from turbot liver tissues (Figure 1a). The Mg concentration in muscle tissue is correlated negatively with the Mg from liver tissues, while the Mg concentration in liver tissue is correlated negatively with the Na in both analyzed tissues (Figure 1a).

Additionally, negative correlations are observed between K in liver and Na in liver, respectively Na in turbot muscle tissues (Figure 1a).

Other studies [41] reported significant positive correlations between Mn-Cr, As-Mn, As-Cu, Zn-Fe, Zn-Mn, and Zn-Cd, while significant negative correlations were reported between Mn-Ni, As-Fe, As-Ni, Zn-Cu, Zn-Ni, and Hg-Cr in fish muscle tissues, when considering the following fish species: *Scardinus knezevici*, *Alburnus scoranza*, *Cyprinus carpio*, *Rutilus prespensis*, *Anguilla anguilla* and *Perca fluviatilis*. The correlations are different compared to those recorded in the present study, for analyzed element from turbot muscle tissues. This can be due to a difference in terms of ecology and habitat between turbot and the aforementioned fish species. Pelagic and benthic fish had different pathways for metal accumulation. For instance, some authors [42] observed in their study that fish feeding on pelagic prey accumulated higher concentration of Fe and Zn. On the other hand, benthic fish accumulated higher concentrations of Cd, Ni, Pb, Co, As, and Sn [42]. Additionally, other authors [43] reported that *Sardinella* sp. accumulated high metals concentrations, due to feeding ecology (phytoplankton with high Zn and Cu concentration).

Additionally, the habitat plays an important role in fish metals accumulation. Some authors [42] observed that in the aquatic ecosystem subjected to high anthropogenic metals input, fish from the benthic environment accumulated higher concentrations of metals, whereas, in an unaffected aquatic ecosystem, the bioaccumulation was associated with pelagic fish.

Several authors have reported significant correlations between different heavy metals that were detected in the environmental matrix (water–sediments–aquatic organisms). Thus, other authors [44] pointed out a positive correlation between Cu, Zn, Cd, and As, in the water of Chonglingjiang River, China. Another research study [45] reported positive correlation between Cu and Zn in the water of Pearl River Estuary and a very strong correlation between Cu and Cd in the river sediments. Rajkowska [46] observed a positive correlation between the concentration of Fe in the water matrix and muscle of *Esox lucius* (Liunnaeus, 1758), a freshwater fish.

The correlations between the elements, which occurred in fish tissues, are also influenced by physiological processes. Thus, the accumulation of some metals in fish is influenced by the presence of other metals and interactions between them are related to their different binding affinities to different organs [47]. For instance, Ca has strong antagonistic effect on Cd accumulation and toxicity [48]. High calcium concentrations prevent cadmium uptake by competing at uptake sites [49]. Cadmium ions enter the chloride cells in the gills through calcium channels [47,48,49]. In this context, when considering that physiological processes in the fish body are complex and difficult to be quantified, the present paper utilizes machine learning techniques to identify possible patterns in the interactions between the elements in the turbot’s body.

### 2.2. Predictive Models

There are many scientific studies proving the efficiency of linear methods for heavy metal-sensing and interdependency determination [50,51,52,53,54]. Thus, some authors used various analytical methods in their study [50], including stepwise multi-linear regression and partial least squares regression to model metal content level in soil and vegetation. Other authors [51] investigated the concentrations of chromium (Cr), nickel (Ni), copper (Cu), zinc (Zn), cadmium (Cd), and lead (Pb) in vegetables, corresponding cultivated soils and irrigation waters from 36 open sites in high natural background area of Wuzhou, South China. Redundancy analysis, Spearman’s rho correlation analysis, and multiple regression analysis were adopted in order to evaluate the contributions of impacting factors on metal contents in the edible parts of vegetables.

In some studies’ [52] research, soil and crop samples were collected from agricultural areas in the Yangtze River Delta region. The concentration of Zn and Cd in crop grains could be well predicted according to the stepwise multiple linear regression models, which could help to quantitatively evaluate the ecologic risk of heavy metal accumulation in crops in the study area.

A large study targeting heavy metal presence based on linear regression analysis was conducted by other authors [53]. In this work, the authors analyzed the changes in the content of heavy metals in 118 water bodies in Kazakhstan, between 1997–2017, determining the relationship between the content of heavy metals and the level of anthropogenic load to reveal natural factors affecting the accumulation of heavy metals in the water. Regression analysis revealed the leading role of pH in the accumulation of heavy metals in water bodies and the fact that an increase in anthropogenic load in the direction from mountainous areas to plains led to the additional enrichment of water bodies with heavy metals.

Some researches [54] use linear stepwise regression models for investigating a problem of food safety determined by the bivalve mollusks of *Anadara granosa*, as dangerous metals in the environment may accumulate in their soft tissue. His research aimed to analyze the seasonal pattern (Indonesian east, intermediate, and west monsoon season) of heavy metals (Cr, Co, As, Cd, Hg, and Pb) accumulation factor in the soft tissue. His linear models showed that only Cr and Pb had a significant relation (*p* < 0.05) between their accumulation in environment and in the molluscs.

The stepwise procedure for determining the relevant parameters to be embedded in the model is emphasized by some authors [55]. After examining relevant papers that were published by three leading ecological and behavioral journals, he shows that the use of this technique remains widespread: from 65 papers in which a multiple regression approach was used, 57% of studies used the stepwise regression method.

#### 2.2.1. The First Group MLR Models

A number of 9 MLR models were identified after processing the first group dataset (model 1–9)—for the group description see 3.4. Thus, four MLR models (model 1, 3, 5, 7) were identified for muscle tissue elements (Ca, Fe, Mg, Na) and five MLR models for liver tissues elements (Cu, K, Na, Zn) (2, 4, 6, 8a, 8b).

The first MLR model (model 1) determines the concentration of Ca in turbot muscle tissues, based on K and Mn concentration in muscle tissues, Ca and Cd concentration in liver, and, also, turbot weight. The model explains 88.05% of the variance of Ca in turbot muscle tissues. Additionally, the value of predicted R-sq is close to the R-sq value, a situation that indicates a good model performance (model 1). However, the S-value is high, a situation that indicates that the model does not achieve the best degree of precision (model 1). The codded coefficients permit us to identify the variable with the largest impact on model response. Thus, for model 1, the value of Ca in liver has the strongest influence on the resulted concentration of Ca in turbot muscle tissues.

The second model determines the concentration of Cu in turbot liver tissues, based on Cu concentration in muscle tissues, and Fe, Ni, and Zn concentrations in the liver (model 2). The model explains 77.73% of the variance of Cu in turbot liver tissues. The model indicates a good performance (predicted R-sq value is close to R-sq), a situation also revealed by low S-value that indicates a high degree of precision (model 1). By analyzing the codded coefficients of model 2, it can be stated that the value of Zn and Ni in liver has the strongest influence on the resulted concentration of Cu in turbot liver tissues.

The third model determines the concentration of Fe in turbot muscle tissues, based on Ni concentration in muscle tissues and As, Mg, and Mn concentration in liver (model 3). The model explains 83.86% of the variance of Fe in turbot muscle tissues. The model indicates a good performance (predicted R-sq value is close to R-sq), a situation also revealed by the low S-value that indicates a high degree of precision (model 3). By analyzing the codded coefficients of model 3, it can be stated that the values of Mg in liver and Ni in muscle tissues have the strongest influence on the resulted concentration of Fe in turbot muscle tissues.

The fourth model determines the concentration of K in turbot liver tissues, based on Mg concentration in muscle tissues and Ca concentration in the liver (model 4). The model explains 74.71% of the variance of K in turbot liver tissues. The high S-value indicates that the model does not achieve the best degree of precision (model 4). By analyzing the codded coefficients of model 4, it can be stated that the values of Ca in liver tissues has the most significant impact over the dependent variable.

The fifth model determines the concentration of Mg in turbot muscle tissues, based on Na and Ni concentration in muscle. The model explains 71.21% of the variance of Fe in turbot muscle tissues. The model indicates a good performance (predicted R-sq value is close to R-sq), situation revealed also by relatively low S-value that indicates good precision (model 5). By analyzing the codded coefficients of model 5, it can be stated that Ni in muscle tissues has the most significant impact over the dependent variable—Mg in turbot muscle tissues.

The sixth model determines the concentration of Na in turbot liver tissues, based on Cd, Cu, and Mg concentration in liver tissues. The model explains 80.25% of the variance of Na in turbot liver tissues. The model indicates a good performance (predicted R-sq value is close to R-sq), situation revealed also by low S-value that indicates a high degree of precision (model 6). By analyzing the codded coefficients of model 6, it can be stated that Mg in liver tissues has the most significant impact on Na in turbot liver tissues.

The seventh MLR model determines the concentration of Na in turbot muscle tissues, based on Mg concentration in muscle tissues and Ca concentration in liver. The model explains 91.71% of the variance of Na in turbot muscle tissues. The model indicates a very good performance (predicted R-sq value is close to R-sq), situation revealed also by low S-value that indicates a considerable high degree of precision (model 7). The value of codded coefficients presented for model 7 indicates that Ca in liver tissues has the most significant impact over the dependent variable.

The last two MLR models (model 8a and model 8b) determine the concentration of Zn in turbot liver tissues, based on Cu, K and Mn concentration in muscle tissues and Cu and K concentration in liver (model 8a), respectively, on the Cu and Mg concentration in liver and Mn in muscle (model 8b). The models explain 83.33%, respectively, 79.61% of Zn in turbot liver tissues. When considering the predicted R-sq value, the R-sq, and the S-value, it can be stated that model 8b has a higher degree of precision, as compared to model 8a. The codded coefficients of both models reveal that Cu in liver tissues has the most significant impact over the dependent variable.

**Figure A1:**
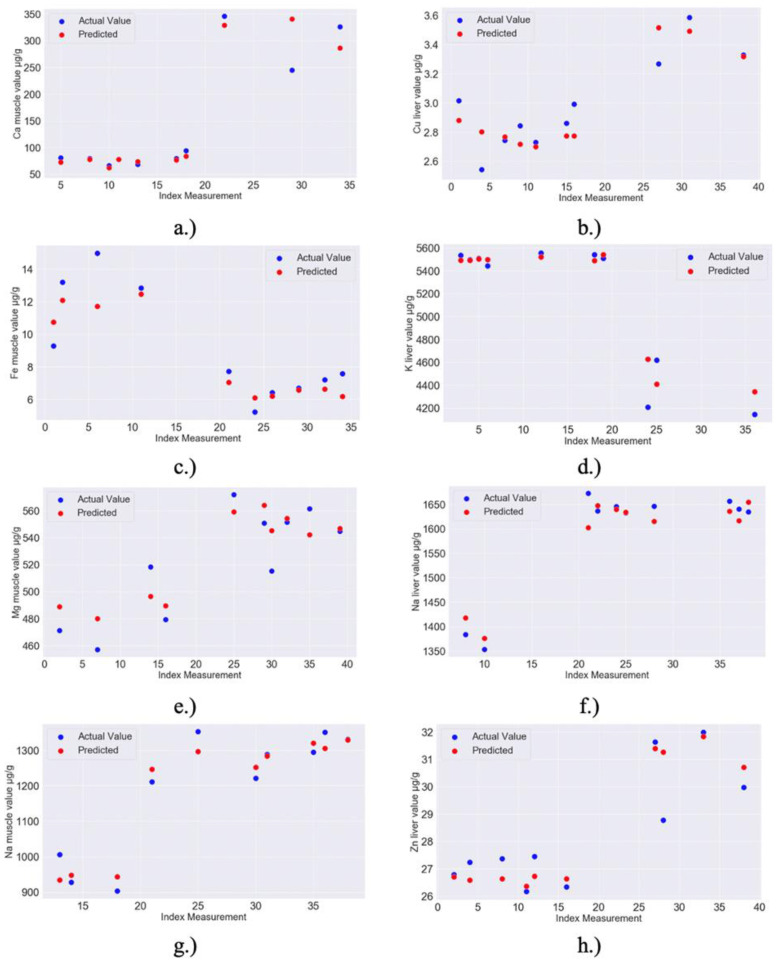
Prediction of heavy metal concentration for turbot liver and muscle tissues—actual values vs. predicted value of RF models for the first group MLR models, described in Section 2.2.1. (models 1–8b) ((**a**). prediction for Ca concentration in muscle; (**b**). prediction for Cu concentration in liver; (**c**). prediction for Fe concentration in muscle; (**d**). prediction for K concentration in liver; (**e**). prediction for Mg concentration in muscle; (**f**). prediction for Na concentration in liver; (**g**). prediction for Na concentration in muscle; (**h**). prediction for Zn concentration in liver).

Every parameter that was explained through a relevant MLR model was also predicted by a RF model in order to aggregate how many times each heavy metal parameter contributed as an important predictor in a random forest model (Table A1 and Figure A1. This way, it was possible to provide a complete overview over each of the heavy metal parameters’ importance, from a RF perspective—how important is each parameter in the prediction models. Thus, it resulted that Ca and Na elements are the most important for the RF prediction version of the models described below (model 1–8).

*Model 1*

MLR: Ca muscle = 236 + 648 Cd liver + 567 Mn muscle + 4.437 Ca liver − 0.0719 K muscle − 120.9 Turbot Weight (S = 45.96, R-sq = 88.05%, R-sq (adj) = 86.29%, R-sq (pred) = 81.64, 10-fold S = 51.14, 10-fold R-sq = 82.59%).

Variance analysis: Cd liver (*p* = 0.06), K muscle (*p* = 0.04), turbot weight (*p* = 0.03), Mn muscle, Ca liver (*p* < 0.01).

Codded coefficients (after standardization): 176.84 (*p* < 0.01) for constant, 14.94 (*p* = 0.06) for Cd liver, 32.81 (*p* < 0.01) for Mn muscle, 127.87 (*p* < 0.01) for Ca liver, −17.62 (*p* = 0.04) for K muscle, and −18.12 (*p* = 0.028) for Turbot weight.

Most important variable in RF model: Ca concentration in liver tissues (Table A1).

*Model 2 (Zn liver)*

MLR: Cu liver = 4.17 + 0.09 Zn liver − 8.98 Ni liver − 0.02 Fe liver − 7.00 Cu muscle (S = 0.21, R-sq = 77.73%, R-sq (adj) = 75.18%, R-sq (pred) = 71.88, 10-fold S = 0.22, 10-fold R-sq = 71.81%).

Variance analysis: Zn liver (*p* < 0.01), Ni liver (*p* < 0.01), Fe liver (*p* < 0.01), Cu muscle (*p* = 0.02).

Codded coefficients (after standardization): 3.10 (*p* < 0.01) for constant, 0.23 (*p* < 0.01) for Zn liver, −0.28 (*p* < 0.01) for Ni liver, −0.19 (*p* < 0.01) for Fe liver, −0.08 (*p* = 0.03) for Cu muscle.

Most important variable in RF model: Zn concentration in liver tissues (Table A1).

*Model 3*

MLR: Fe muscle = −14.89 + 0.16 As liver + 2.29 Mn liver + 44.27 Ni muscle + 0.04 Mg liver (S = 1.56, R-sq = 83.86%, R-sq (adj) = 82.02%, R-sq (pred) = 78.75, 10-fold S = 1.74, 10-fold R-sq = 77.25%).

Variance analysis: As live (*p* = 0.03), Mn liver (*p* = 0.03), Ni (*p* < 0.01), Mg liver (*p* < 0.01).

Codded coefficients (after standardization): 9.14 (*p* < 0.01) for constant, 0.62 (*p* = 0.03) for As liver, 0.62 (*p* = 0.03) for Mn liver, 1.34 (*p* < 0.01) for Ni muscle, 3.58 (*p* < 0.01) for Mg liver.

Most important variable in RF model: Na concentration in muscle tissues (Table A1).

*Model 4*

MLR: K liver = 5054.00 − 27.31 Ca liver + 4.21 Mg muscle (S = 387.34, R-sq = 74.71%, R-sq (adj) = 73.34%, R-sq (pred) = 70.43, 10-fold S = 404.55, 10-fold R-sq = 70.18%).

Variance analysis: Ca liver (*p* < 0.01), Mg muscle (*p* = 0.04).

Codded coefficients (after standardization): 4889.00 (*p* < 0.01) for constant, −787.00 (*p* < 0.01) for Ca liver, 198.80 (*p* = 0.04) for Mg muscle.

Most important variable in RF model: Na concentration in liver tissues (Table A1).

*Model 5*

MLR: Mg muscle = 329.20 + 0.21 Na muscle − 387.00 Ni muscle (S = 26.02, R-sq = 71.21%, R-sq (adj) = 69.66%, R-sq (pred) = 66.12, 10-fold S = 28.12, 10-fold R-sq = 63.68%).

Variance analysis: constant (*p* < 0.01), Na muscle (*p* < 0.01), Ni muscles (*p* = 0.01).

Codded coefficients (after standardization): 518.08 (*p* < 0.01) for constant, −11.74 (*p* < 0.01) for Na muscle, 41.85 (*p* = 0.01) for Ni muscles.

Most important variable in RF model: Na concentration in muscle tissues (Table A1).

*Model 6*

MLR: Na liver = 1717.00 − 1037.00 Cd liver + 109.40 Cu liver − 1.03 Mg liver (S = 69.53, R-sq = 80.25%, R-sq (adj) = 78.61%, R-sq (pred) = 75.11, 10-fold S = 76.19, 10-fold R-sq = 73.65%).

Variance analysis: Cd liver (*p* = 0.03), Cu liver (*p* < 0.01), and Mg liver (*p* < 0.01).

Codded coefficients (after standardization): 1511.60 (*p* < 0.01) for constant, −23.90 (*p* = 0.04) for Cd liver, 46.30 (*p* < 0.01) for Cu liver, −97.10 (*p* < 0.01) for Mg liver.

Most important variable in RF model: Ca concentration in liver tissues (Table A1).

*Model 7*

MLR: Na muscle = 177.00 + 5.54 Ca liver + 0.90 Mg muscle (S = 59.79, R-sq = 91.71%, R-sq (adj) = 91.27%, R-sq (pred) = 90.03, 10-fold S = 62.61, 10-fold R-sq = 90.18%).

Variance analysis: Ca liver (*p* < 0.01), Mg muscle (*p* = 0.01).

Codded coefficients (after standardization): 1116.55 (*p* < 0.01) for constant, 159.50 (*p* < 0.01) for Ca liver, 42.40 (*p* < 0.01) for Mg muscle.

Most important variable in RF model: Na concentration in muscle tissues (Table A1).

*Model 8a*

MLR: Zn liver = 11.90 − 0.01 Mg liver + 0.002 K muscle + 33.2 Cu muscle + 2.70 Cu liver − 7.01 Mn muscle (S = 1.09, R-sq = 83.33%, R-sq (adj) = 80.88%, R-sq (pred) = 74.76, 10-fold S = 1.25, 10-fold R-sq = 74.37%).

Variance analysis: constant (*p* = 0.05), Mg liver (*p* < 0.01), K muscle (*p* = 0.06), Cu muscle (*p* = 0.03), Cu liver (*p* < 0.01), and Mn muscle (*p* = 0.04).

Codded coefficients (after standardization): 28.63 (*p* < 0.01) for constant, −1.06 (*p* < 0.01) for Mg liver, 0.38 (*p* = 0.06) for K muscle, 0.40 (*p* = 0.04) for Cu muscle, 1.14 (*p* < 0.01) for Cu liver, −0.41 (*p* = 0.05) for Mn muscle.

*Model 8b*

MLR (model using less predictors): Zn liver = 25.31 + 2.94 Cu liver − 7.49 Mn muscle − 0.01 Mg liver (S = 1.17, R-sq = 79.61%, R-sq (adj) = 77.91%, R-sq (pred) = 74.23, 10-fold S = 1.29, 10-fold R-sq = 72.59%).

Variance analysis: Cu liver (*p* < 0.01), Mn muscle (*p* = 0.04), and Mg liver (*p* < 0.01).

Codded coefficients (after standardization): 28.63 (*p* < 0.01) for constant, 1.24 (*p* < 0.01) for Cu liver, −0.43 (*p* = 0.04) for Mn muscle, −0.98 (*p* < 0.01) for Mg liver.

Most important variable in RF model: Ca concentration in liver tissues (Table A1).

It can be observed that, except for model 1, the rest of the models are based on element to element relationships. However, fish size and weight are important factors that influence metal accumulation in tissue [56,57]. The accumulation degree is also highly dependent on fish species. For instance, some authors [57] obtained in their study no significant relationship between fish size and Cu concentration.

Grass carp had a positive correlation between its size and concentration of Zn, Pb and Cd [57]. On the other hand, catfish registered a negative correlation between size and Hg concentration, while a positive correlation between these variables was registered in grass carp and common carp [57]. Similar results were reported by other authors [56], who obtained positive correlation between fish weight and the concentration of Cd and Pb, respectively.

#### 2.2.2. The First Group Non-Linear Tree-Based RF Prediction Models

Non-linear models were tested for determining possible models among the parameters that did not register linear correlation between each other. The RF represents one of the best available methods that can be used to assess both a prediction algorithm and feature importance inside the model.

Some of the authors [58,59] identify that the RF, as developed in [60], is one of the most successful machine (statistical) learning algorithms for practical applications. There are many scientific fields where RF has been applied: agriculture [61], ecology [62], land cover classification [63], remote sensing [64,65], wetland classification [66], bioinformatics [67], as well as biological and genetic association studies [68] and genomics [69].

A number of 12 RF models were identified after processing the dataset (model 9–20). Thus, six RF models (model 9, 12, 14, 16, 19, 20) were identified for muscle tissues elements (As, Cd, Cu, K, Mn, Zn) and six RF models (model 10, 11, 13, 15, 17, 18) for liver tissues elements (As, Ca, Cd, Fe, Mg, Mn). The regressors of the above models (model 9–20) are presented in the Appendix A (Table A2).

*Model 9*

RF model: As muscle–Feature importance: 0.17 for Cd liver, 0.10 for K muscle, 0.06 for K liver, 0.05 for Zn muscle, 0.04 for Ca liver; Model Accuracy: 85.40% (MAPE = 14.60%).

*Model 10*

RF model: As liver–Feature importance: 0.14 for Ni muscle, 0.12 for Fe muscle, 0.07 for Zn liver, 0.06 for Na muscle, 0.03 for Mn muscle; Model Accuracy: 77.30% (MAPE = 22.70%).

*Model 11*

RF model: Ca liver–Feature importance: 0.04 for Na liver, 0.02 for Ca muscle, 0.02 for Zn liver, 0.02 for Na muscle, 0.01 for Ni liver; Model Accuracy: 97.40% (MAPE = 2.60%).

*Model 12*

RF model: Cd muscle–Feature importance: 0.07 for Mg muscle, 0.05 for Ca liver, 0.03 for Zn liver, 0.03 for As muscle, 0.03 for Ca muscle; Model Accuracy: 98.44% (MAPE = 1.56%).

*Model 13*

RF model: Cd liver–Feature importance: 0.23 for K muscle, 0.06 for Cu muscle, 0.06 for Ca liver, 0.02 for ni liver, 0.02 for Zn liver; Model Accuracy: 85.86% (MAPE = 14.14%).

*Model 14*

RF model: Cu muscle–Feature importance: 0.14 for Mg muscle, 0.07 for Fe liver, 0.07 for K liver, 0.05 for Na muscle, 0.03 for Cd muscle; Model Accuracy: 96.25% (MAPE = 3.75%).

*Model 15*

RF model: Fe liver–Feature importance: 0.02 for Ni muscle, 0.01 for K muscle, <0.01 (0.007) for Mn liver, <0.01 (0.005) for Cd muscle, <0.01 (0.003) for Cu liver; Model Accuracy: 94.37% (MAPE = 5.63%).

*Model 16*

RF model: K muscle–Feature importance: 0.13 for Zn muscle, 0.04 for Mg liver, 0.03 for As muscle, 0.03 for Zn liver, 0.01 for Cu muscle; Model Accuracy: 98.06% (MAPE = 1.94%).

*Model 17*

RF model: Mg liver–Feature importance: 0.07 for Na liver, 0.06 for K liver, 0.05 for Fe muscle, 0.04 for Ca muscle, 0.03 for Zn liver; Model Accuracy: 98.14% (MAPE = 1.86%).

*Model 18*

RF model: Mn liver–Feature importance: 0.21 for Fe muscle, 0.11 for Cu liver, 0.04 for As liver, 0.04 for Ni muscle, 0.03 for Mg liver; Model Accuracy: 80.91% (MAPE = 19.09%).

*Model 19*

RF model: Mn muscle–Feature importance: 0.56 for As liver, 0.11 for Cd liver, 0.07 for As muscle, 0.06 for Mn liver, 0.04 for K muscle; Model Accuracy: 94.96% (MAPE = 5.04%).

*Model 20*

RF model: Zn muscle–Feature importance: 0.05 for Ca liver, 0.03 for K muscle, 0.03 for Zn liver, 0.02 for K muscle, 0.02 for Ca muscle; Model Accuracy: 92.13% (MAPE = 7.87%).

The RF technique was applied on all the dataset variables. However, the models that displayed the highest accuracy, respectively, the lowest MAPE value were selected and are presented above (model 9–20). After running the feature importance algorithm, we identified the weight of all independent variables. It is important to emphasize that weight reveals the importance of a specific parameter in determining the dependent variable value.

The Cd in liver and K in muscle tissues are the most important parameters for predicting As concentration in muscle tissues (model 9). The model for predicting As concentration in liver tissues identifies Fe and Ni in muscle tissues as the most important parameter (model 10). In predicting Ca concentration in liver tissues (model 11), the most important parameter is Na in liver tissues. The next model, for predicting Cd concentration in muscle tissues (model 12), identifies Mg in the muscle and Ca in the liver of turbot specimens as most important parameters. The 13th model for predicting Cd concentration in liver tissues (model 13) identifies K in muscle as most important variable. Concentration of Cu in muscle tissues (model 14) is mostly dependent on Mg concentration from muscle tissues. The most important parameters for Fe concentration in liver tissues, using RF prediction (model 15) are Ni and K in muscle tissues. The 16th model, for predicting K concentration in muscle tissues, considers Zn concentration in turbot muscle as the most important parameter.

The 17th model, for predicting Mg concentration in liver tissues, considers, as the most important parameters, this RF prediction the K and Na concentration in liver tissues. The prediction of Mn concentration in liver tissues (model 18) is mostly based on Fe concentration in muscle tissues, while, according to the 11th model, the prediction of Mn in muscle tissues is strongly influenced by the As concentration in turbot liver. The next model, for predicting Zn concentration in muscle tissues (model 12), considers Ca in liver tissues to be the most important parameter.

After analyzing the previous models (model 9–20), the resulted relevant parameters were extended with both turbot weight and turbot length variables in order to evaluate the impact of these two variables on the elements prediction by using non-linear tree-based RF prediction models. Thus, a series of new RF prediction models were identified (model 21–32). Appendix A (Table A2) presents the regressors of the following models (model 21–30).

*Model 21*

RF model: As muscle–Feature importance: 0.37 for Cd liver, 0.10 for K liver, 0.1 for K muscle, 0.05 for Turbot length and 0.05 for Ca liver; Model Accuracy: 89.37% (MAPE = 10.63%).

*Model 22*

RF model: As liver–Feature importance: 0.17 for Cu muscle, 0.10 for Turbot weight, 0.09 for Zn muscle, 0.04 for As muscle; Model Accuracy: 75.16% (MAPE = 24.84%).

*Model 23*

RF model: Ca liver–Feature importance: 0.15 for Ni liver, 0.15 for Ca muscle, 0.11 for Na muscle, 0.09 for Na liver, 0.03 for Zn liver; Model Accuracy: 96.64% (MAPE = 3.36%).

*Model 24*

RF model: Cd muscles–Feature importance: 0.30 for Ca liver, 0.24 for Zn liver, 0.18 for Turbot Weight, 0.13 for As muscle, 0.07 for Mg muscle, 0.04 for Ca muscle, 0.01 for Turbot length; Model Accuracy: 98.59% (MAPE = 1.41%).

*Model 25*

RF model: Cd liver–Feature importance: 0.19 for K muscle, 0.14 for Ca liver, 0.1 for Turbot weight; Model Accuracy: 98.59% (MAPE = 1.41%).

*Model 26*

RF model: Cu muscles–Feature importance: 0.51 for Mg muscle, 0.32 for Na muscle, 0.29 for Turbot length, 0.04 for Ni muscle and 0.04 for Turbot weight; Model Accuracy: 97.03% (MAPE = 2.97%).

*Model 27*

RF model: Fe liver–Feature importance: 0.37 for Ni muscle, 0.23 for Cu liver, 0.16 for Cd muscle, 0.04 for Turbot length, 0.01 for Mn liver; Model Accuracy: 94.21% (MAPE = 5.79%).

*Model 28*

RF model: K muscle–Feature importance: 0.37 for Zn muscle, 0.21 for Zn liver, 0.19 for Mg liver, 0.03 for Turbot weight and 0.03 for As muscle; Model Accuracy: 98.27% (MAPE = 1.73%).

*Model 29*

RF model: Mg liver–Feature importance: 0.18 for Ca muscle, 0.11 for K liver, 0.11 for Zn liver, 0.09 for Fe muscle and 0.05 for Na liver; Model Accuracy: 97.91% (MAPE = 2.09%).

*Model 30*

RF model: Mn liver–Feature importance: 0.53 for As liver, 0.41 for Fe muscle, 0.23 for Turbot weight, 0.03 for Turbot length; Model Accuracy: 84.13% (MAPE = 15.87%).

*Model 31*

RF model: Mn muscle–Feature importance: 0.34 for Turbot length, 0.31 for Cd liver, 0.15 for Turbot weight; Model Accuracy: 91.84% (MAPE = 8.16%).

*Model 32*

RF model: Zn muscle–Feature importance: 0.15 for K muscle, 0.15 for Cu liver, 0.03 for Cd liver, and 0.01 for Turbot weight; Model Accuracy: 92.01% (MAPE = 7.99%)

By analyzing the above-mentioned models (model 21–30), it can be observed that the turbot weight is an important variable for predicting Cd in muscle tissues, as well as As and Mn in liver tissues of turbot specimens. Additionally, turbot length is important for predicting Cu and Mn in turbot muscle tissues, if using non-linear tree-based RF prediction techniques.

#### 2.2.3. The Second Group Non-Linear Tree-Based RF Prediction Models

A number of five RF models were identified after processing the second group dataset. All five models (model 33–37) are predicting the micro-elements (Zn, Cd, Fe, Cu, Ni) concentration in muscle tissues of wild turbot specimens using non-linear tree-based RF prediction models. The regressors of these models (model 33–37) are presented in the Appendix A (Table A2).

*Model 33*

RF model: Zn muscle–Feature importance: 0.08 for Mn muscle, 0.07 for Cu muscle, 0.03 for Ni muscle and 0.02 for Fe muscle; Model Accuracy: 88.36% (MAPE = 11.64%).

*Model 34*

RF model: Cd muscle–Feature importance: 1.37 for Fe muscle, 0.04 for Mn muscle and 0.02 for Ni muscle; Model Accuracy: 98.08% (MAPE = 1.92%).

*Model 35*

RF model: Fe muscle–Feature importance: 0.22 for Zn muscle, 0.15 for Ni muscle, 0,11 for Cd muscle, 0.08 for Mn muscle and 0.03 for Cu muscle; Model Accuracy: 88.36% (MAPE = 11.64%).

*Model 36*

RF model: Cu muscle–Feature importance: 2.75 for Ni muscle and 0.10 for Zn muscle; Model Accuracy: 83.19% (MAPE = 16.81%).

*Model 37*

RF model: Ni muscle–Feature importance: 0.10 for Mn muscle, 0.05 for Fe muscle and 0.03 for Cu muscle; Model Accuracy: 85.85% (MAPE = 14.15%).

The second dataset RF models reveals that Mn and Cu are the most important parameters for predicting Zn concentration in wild turbot muscle tissues (model 33). Additionally, the Fe concentration contributes the most in predicting Cd, while Ni is the most important parameter for the prediction of Cu concentration in wild turbot muscle tissues (model 34, 36). The prediction of Fe is mostly based on Zn concentration, while for Ni prediction, the concentration of Mn in wild turbot muscle is the most important (model 35, 37).

#### 2.2.4. The Third Group MLR Models

A single MLR model was identified after processing the third group dataset (model 38). The model determines the concentration of Zn in turbot muscle tissues, based on Cu and Cd concentrations in muscle. The model explains 79.21% of the variance of Zn in turbot muscle tissues. The model indicates a good performance (predicted R-sq value is close to R-sq), a situation also revealed by low S-value that indicates a high degree of precision (model 38). By analyzing the codded coefficients of model 38, it can be stated that the value of Cd has the strongest influence on the resulted concentration of Zn in turbot muscle tissues.

*Model 38*

MLR: Zn muscle = −0.19 + 4.06 Cu muscle + 380.3 Cd muscle (S = 3.91, R-sq = 79.21%, R-sq (adj) = 78.30%, R-sq (pred) = 71.83, 10-fold S = 4.39, 10-fold R-sq = 72.11%).

Variance analysis: Cu (*p* < 0.01), Cd (*p* < 0.01).

Codded coefficients (after standardization): 4.50 (*p* < 0.01) for Cu, 5.61 (*p* < 0.01) for Cd.

#### 2.2.5. The Fourth Group Non-Linear Tree-Based RF Prediction Models

A number of 5 RF models were identified after processing the fourth group dataset. All five models (model 39–43) are predicting the micro-elements (Cd, Cu, Fe, Ni, Zn) concentration in muscle tissues of wild and aquaculture turbot specimens using non-linear tree-based RF prediction models. Appendix A (Table A2) presents the regressors of these models (model 39–43).

*Model 39*

RF model: Cd muscle–Feature importance: 0.06 for Fe muscle; Model Accuracy: 95.47% (MAPE = 4.53%).

*Model 40*

RF model: Fe muscle–Feature importance: 0.28 for Zn muscle, 0.23 for Cu muscle, 0.21 for Ni muscle, 0.07 for Mn muscle, 0.06 for Cd muscle; Model Accuracy: 81.44% (MAPE = 18.56%).

*Model 41*

RF model: Cu muscle–Feature importance: 0.27 for Ni muscle; Model Accuracy: 92.75% (MAPE = 7.25%).

*Model 42*

RF model: Zn muscle–Feature importance: 0.25 for Mn muscle, 0.13 for Ni muscle, 0.11 for Fe muscle; Model Accuracy: 87.79% (MAPE = 12.21%).

*Model 43*

RF model: Ni muscle–Feature importance: 0.59 for Mn muscle, 0.22 for Zn muscle, 0.03 for Cd muscle; Model Accuracy: 83.61% (MAPE = 16.39%).

The fourth dataset prediction models (model 39–43) reveals that the prediction of Cd is based on Fe concentration (model 39), while the prediction of Cu is based on Ni concentration (model 41). Additionally, Zn, Cu, and Ni are the most important in predicting Fe concentration in wild and aquaculture turbot muscle tissue (model 40). The prediction of Zn and Ni concentrations are mostly based on Mn concentration (model 42, 43).

**Figure A2:**
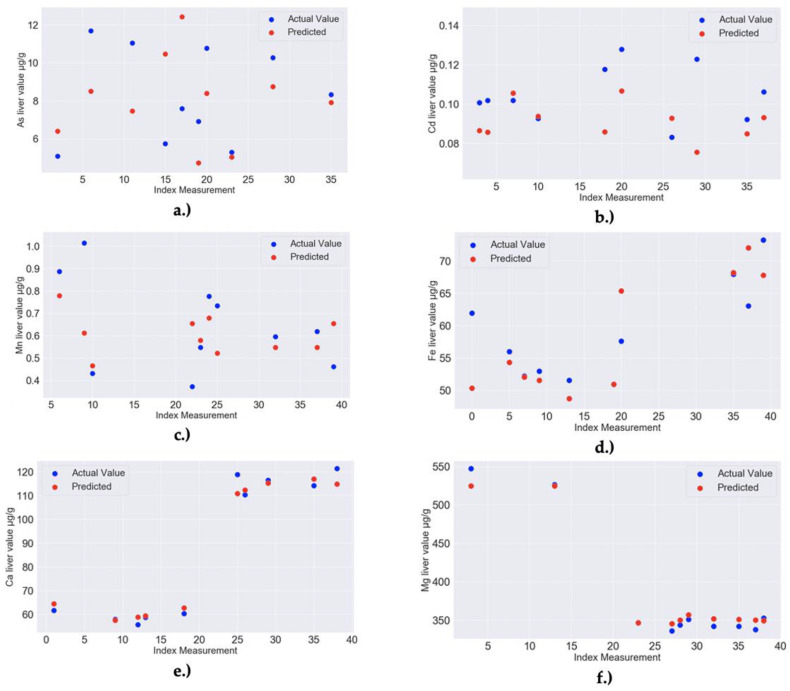
Prediction of heavy metal concentration for turbot liver tissues—actual values vs. predicted value for first group non-linear tree-based RF prediction models, described in Section 2.2.2. ((**a**). prediction for As concentration; (**b**). prediction for Cd concentration; (**c**). prediction for Mn concentration; (**d**). prediction for Fe concentration; (**e**). prediction for Ca concentration; (**f**). prediction for Mg concentration).

**Figure A3:**
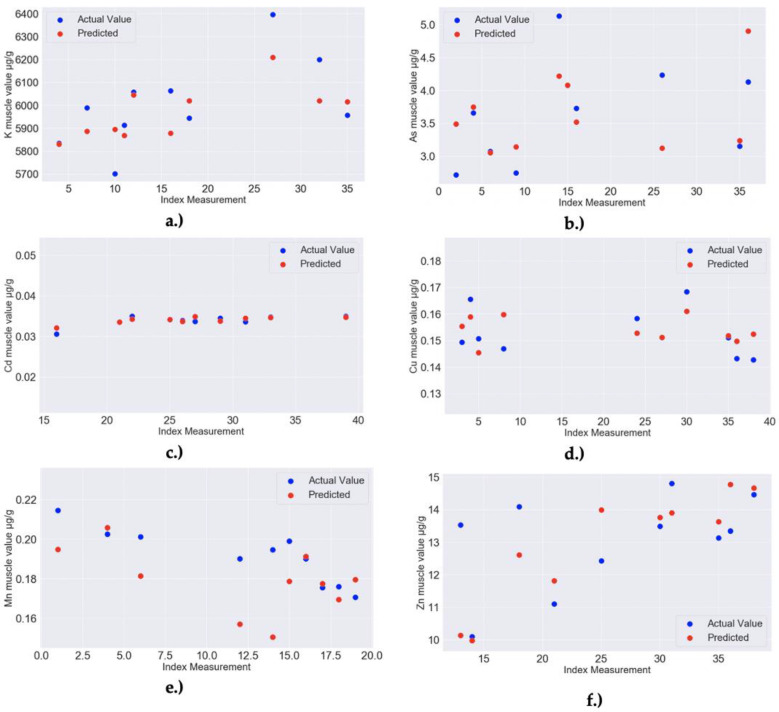
Prediction of heavy metals concentration for turbot muscle tissues—actual values vs. predicted value for first group non-linear tree-based RF prediction models, described in Section 2.2.2. ((**a**). prediction for K concentration; (**b**). prediction for As concentration; (**c**). prediction for Cd concentration; (**d**). prediction for Cu concentration; (**e**). prediction for Mn concentration; (**f**). prediction for Zn concentration).

**Figure A4:**
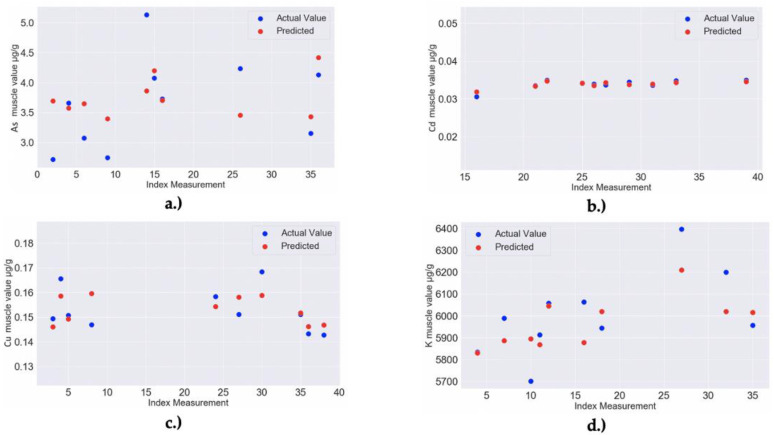

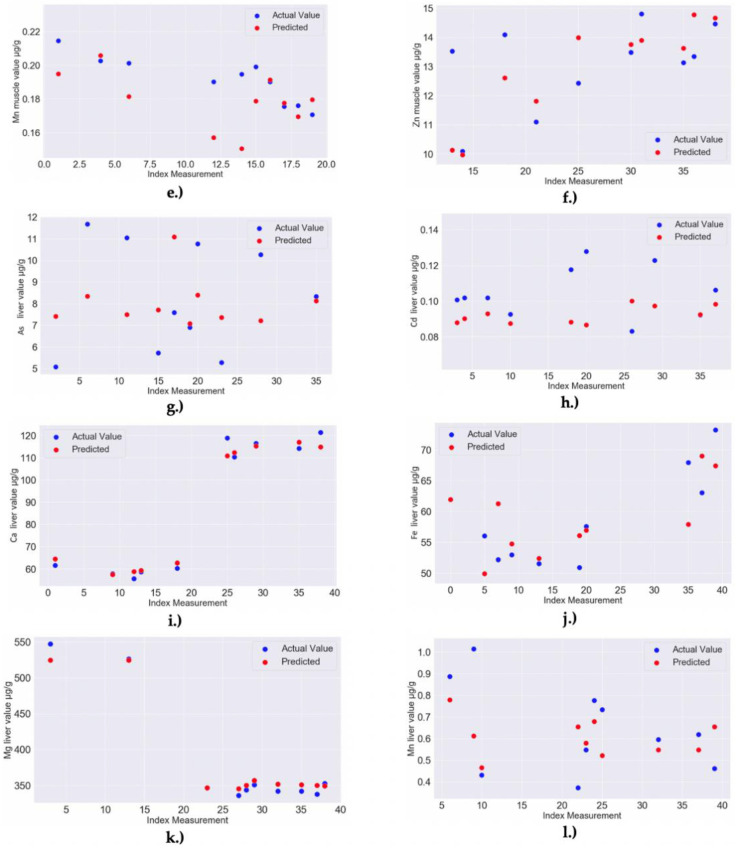
Prediction of heavy metal concentration for turbot muscle and liver tissues—actual values vs. predicted value for first group non-linear tree-based RF prediction models 21–30. ((**a**). prediction for As concentration in muscle; (**b**). prediction for Cd concentration in muscle; (**c**). prediction for Cu concentration in muscle; (**d**). prediction for K concentration in muscle; (**e**). prediction for Mn concentration in muscle; (**f**). prediction for Zn concentration in muscle; (**g**). prediction for As concentration in liver; (**h**). prediction for Cd concentration in liver; (**i**). prediction for Ca concentration in liver; (**j**). prediction for Fe concentration in liver; (**k**). prediction for Mg concentration in liver; (**l**). prediction for Mn concentration in liver).

**Figure A5:**
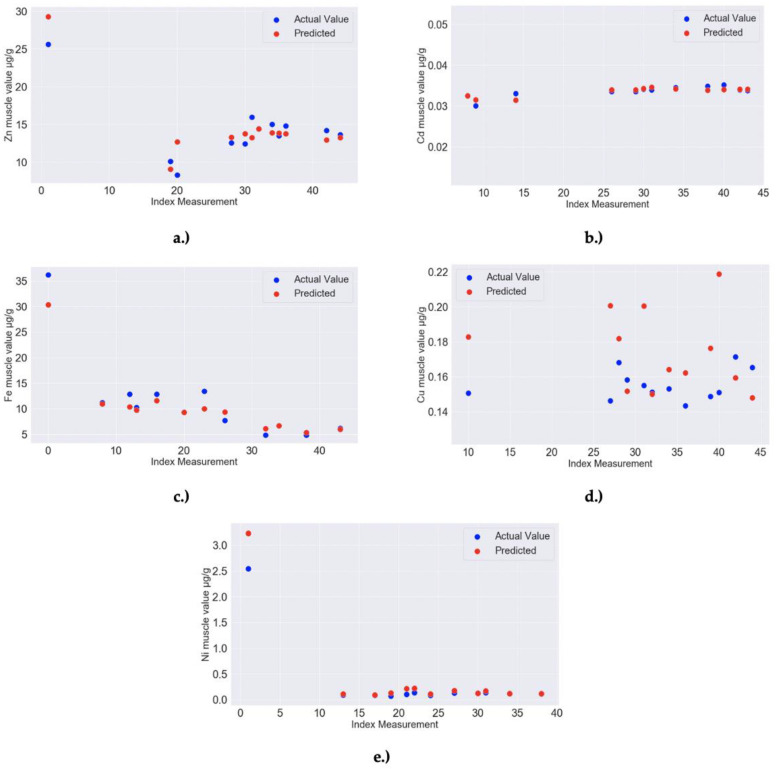
Prediction of heavy metals concentration for turbot muscle tissues—actual values vs. predicted value for second dataset group—Section 2.2.3. ((**a**). prediction for Zn concentration; (**b**). prediction for Cd concentration; (**c**). prediction for Fe concentration; (**d**). prediction for Cu concentration; (**e**). prediction for Ni concentration).

**Figure A6:**
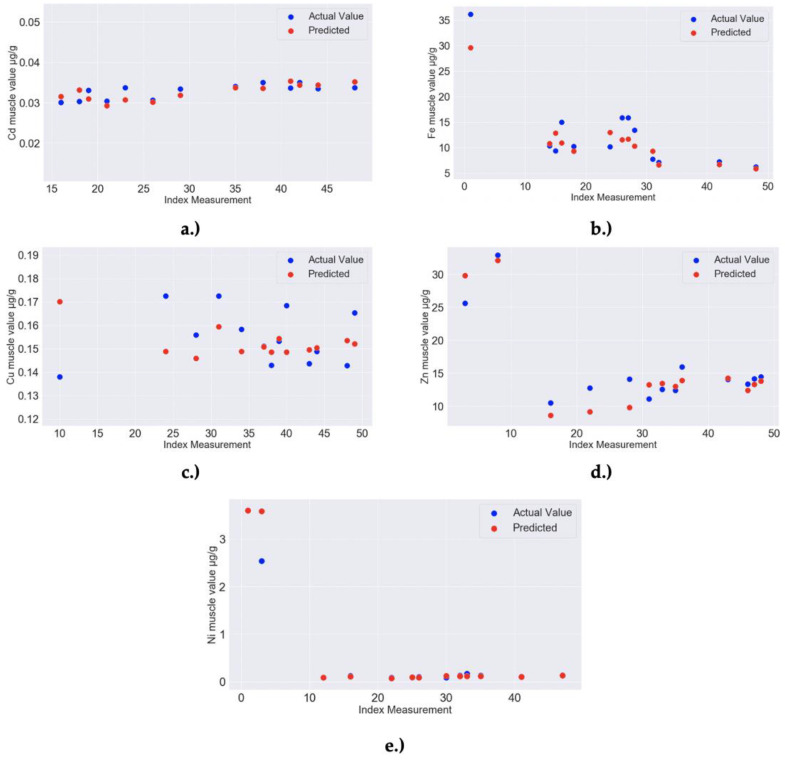
Prediction of heavy metals concentration for turbot muscle tissues—actual values vs. predicted value for fourth dataset group—Section 2.2.5 ((**a**). prediction for Cd concentration; (**b**). prediction for Fe concentration; (**c**). prediction for Cu concentration; (**d**). prediction for Zn concentration; (**e**). prediction for Ni concentration).

Figure A1, Figure A2, Figure A3, Figure A4, Figure A5 and Figure A6 graphically depict how precise the models predicted the values for the test dataset points, which is data never seen previously by the model and not used for model training. Sample measurements dataset contains records composed of all the analyzed parameters, with each record having an index number, that is an integer ranging from 0 to the maximum number of records. A part of the index points (20%) were used as test datapoints for model validation. Therefore, the Figure A1, Figure A2, Figure A3 and Figure A4 compare actual point values with the predicted values, for first group RF models, Figure A5 for the 2nd group and Figure A6 for the 4th group. If for some index points there is only one dot appearing on the chart, that means the prediction value was extremely close to the actual value, with an almost inexistent difference between the two values.

#### 2.2.6. The Fifth Group MLR Models

In terms of elaborating a predictive model that is based on Pb, Cd, and As concentration in turbot muscle tissues, based on the dataset corresponding to the fifth group, it can be concluded that no models were found for predicting Cd and As. However, if As is introduced along with Cd, the R-sq value rises to 47.6% (model 44). This reveals that As explains part of the variance of the Pb dependent variable, but, most probably, the results indicate a low performance due to small number of samples from the fifth group dataset.

*Model 44*

MLR: Pb muscle = −0.148 + 2.74 Cd muscle + 0.264 As muscle (S = 0.20, R-sq = 47.76%, R-sq (adj) = 30.34%, R-sq (pred) = 0.00%, 10-fold S = 0.31, 10-fold R-sq = 0.00%).

Variance analysis: Cd muscle (*p* = 0.24), As muscle (*p* = 0.06).

Codded coefficients (after standardization): constant = 0.24 (*p* = 0.01), Cd muscle = 0.12 (*p* = 0.24), As muscle = 0.21 (*p* = 0.06).

#### 2.2.7. Feature Importance Overview

The following table displays a general overview over the feature importance of the parameters in the first group dataset, respectively, the number of times that each feature appeared as a significant predictor in a model, assessing how many times a specific heavy metal was involved as an important parameter in all developed models. The reason why the parameter importance aggregation was performed only on the first group dataset is related to the fact that first group dataset contains all study parameters. Therefore, considering all RF models developed based on the first group dataset (Table 1), it can be concluded that Ca, K, Zn, and Mg were the most important elements used by the RF models in order to predict the other parameters available in the dataset.

It is well known that fish muscle is not an active tissue involved in heavy metal accumulation, and different fish species contain different concentrations in their muscle [70]. The older fish will accumulate higher concentrations of heavy metals due to long life span. However, the accumulation occurs in target organs that are involved in organism detoxification (such as liver and kidneys), and less in the muscle tissues.

All the analyzed metals can induce toxicity; however, only Cd, Pb and Hg are regulated by the European Law, in terms of concentration in fish meat, due to their high toxicity risk.

The European Law (Directive 2006/1881/EC) [71] regulates the concentration of three metals with the highest toxicity potential: Hg, Cd, and Pb, respectively. The maximum level in fish muscle is 0.3 mg kg^−1^ wet weight for Pb, 0.05 mg kg^−1^ wet weight for Cd, and 0.5 mg kg^−1^ wet weight for Hg. According to the Commission, concentrations within this range are toxicologically acceptable.

Therefore, the dataset that was used for elaborating the models presented in current research did contain some values of Cd [72,73] and Pb [74,75,76] over the maximum levels that were regulated by European Law (Directive 2006/1881/EC).

In the case of Ni concentration, according to WHO (World Health Organization) in most foodstuff the Ni content is less than 0.5 mg kg^−1^. The provisional tolerable daily intake (PTDI) of Ni based on the lowest observed adverse effect level (LOAEL) is 12.0 µg/kg body weight (WHO, 2007) [77].

The Institute of Medicine (2001) recommended 10 mg/day copper as a tolerable upper intake level (UL) for adults from foods and supplements [78]. The UL for adults is 45 mg/day of iron, a level that is based on gastrointestinal distress as an adverse effect [79]. The UL for adults is 40 mg/day of Zn, a value based on reduction in erythrocyte copper-zinc superoxide dismutase activity (Institute of Medicine, 2001). A UL of 11 mg/day manganese was set for adults based on a no-observed-adverse-effect level for Western diets (Institute of Medicine, 2001). Arsenic is found in high concentration mainly in marine products, in the organic form (arsenobetaine), which is not toxic; therefore, no UL was set for arsenic (Institute of Medicine, 2001).

Higher concentrations of different metals in the liver are normal, because of its involvement in organisms’ detox mechanisms. High concentrations of Cu in the liver are related to the natural binding to metallothionein [43]. Regarding Fe, the liver has the physiological role in the of synthesising hemoglobin and red blood cells; therefore, high levels of this element are expected [43].

In the case of macro-elements, according to FAO [80] the average concentration of K in fish muscle is 2780 μg g^−1^. Potassium is the most abundant intracellular ion in fish and it plays many important physiological roles including the maintenance of cellular volume and membrane potentials, the generation of nerve impulses, osmo-and ion-regulation and acid/base balance [81]. Additionally, it is well known that red blood cells contain higher concentrations of potassium compared to the plasma; therefore, it is normal to note high potassium levels in the liver.

If available, information that is related to other parameters, such as temperature, salinity, and pH, can be considered as input data for elaborating heavy metals prediction models. Various studies demonstrated the inverse relationship between water salinity and metal accumulation in aquatic organisms, such as crabs, clams, and fish [82,83,84]. The same phenomenon was noted in the case of water pH and metal accumulation in aquatic biota [85]. In acidic waters, the abundant hydrogen ions bind to the negatively charged surfaces and heavy metals remain without binding sites [86]. Therefore, heavy metals would be present in the soluble form, which are more available for aquatic organisms to accumulate [86]. Low water pH can also cause metals desorption from the sediments and organic ligands, hence increasing meta’ solubility in water [87]. In the case of water temperature and metal accumulation in aquatic organisms, the relationship is directly proportional. According to some authors [88], increased water temperature leads to increased metals accumulation by fish, due to the higher metabolic rate and higher rate of metal uptake.

Similar to most studies, the design of the current study is subject to limitations. Therefore, as mentioned, on the aquatic environment factors, such as water hardness, alkalinity, and pH, influence the uptake process of potentially toxic metals by fish [89]. Cd and Pb are bioavailable for fish to absorb if the aforementioned factors are low.

The presence of Ca and Mg ions in the water determines the precipitation of metals in inorganic compounds, such as carbonates and hydroxides, thus reducing the bioavailability of the metals for fish. The mobility and bioavailability of Cd, Zn and Fe in surface waters is positively influenced by: low pH values, low water hardness, low concentrations of suspended solids, low salinity, and high redox potential [90,91]. The same phenomenon is observed in the case of Pb. The toxicity of Pb decreases in surface waters when the values of pH, alkalinity, and hardness are high. For instance, the formation of lead carbonate and hydroxide occurs in hard waters, which are the least soluble forms of Pb [92].

Pb is the most stable metal in the water environment due to its high resistance to water corrosion [92]. In the case of Fe ions, in water bodies with high pH values, this element precipitates as iron hydroxide, which is poorly soluble in water and, therefore, less available for fish and other aquatic organisms [93]. However, low oxygen levels positively influence the solubility of Fe [92]. The solubility of Cu in water is relatively low and, thus, this metal tends to accumulate in sediments in higher concentrations [94].

Water temperature is another important variable affecting metal and metaloid toxicity. For instance, the bioaccumulation of As in aquatic organisms is doubled at a water temperature of 30 °C, compared to a temperature of 16 °C [95]. Fish bioaccumulation of As is influenced by salinity and marine fish accumulate higher concentrations as compared to freshwater fish [96]. However, the As form in most fish is the organic arsenobetaine, which is relatively non-toxic [22].

In the case of Zn, Anu et al. [97] observed a positive correlation between its bioaccumulation in aquatic organisms and the level of nitrate concentrations in the water.

Therefore, firstly, there are limitations in terms of using fish as bioindicators for environmental pollution. Secondly, the models elaborated in the present paper have limitations, as they are based on data that were provided from the scientific literature obtained by using different heavy metals determinations methods, for turbot exemplars from different environmental habitats.

It should be emphasized that machine learning techniques are data-greedy. They require large volumes of data, coming from as many contexts as possible in order to be able to learn the existing data patterns. The available dataset was appropriate for an algorithm, like random forest, but it would have been insufficient for different approaches, such as neural network/deep learning. Secondly, it is important for the dataset to contain data that come from a variety of sources describing as many cases. For the current research, it would have been better if the external sources would have provided more data as the trained algorithm would have learned more data specific intricacies.

By extending the dataset with more samples, respectively, more parameters, state of the art deep learning algorithms, or support vector machine regression (SVR) could be used in assessing the heavy metal concentrations [98].

If more data would be available, insights regarding heavy metals concentration in liver and muscle tissue could also be obtained by using unsupervised machine learning algorithms, like clustering methods. Through clustering methods, data could be grouped in clusters sharing common properties that could be further investigated. There are plenty of clustering techniques that could be applied: (a) protype based: K-means, ISODATA, Fuzzy K-means, partitioning around medoids, mixture models, and self-organizing maps, (b) density based: grid clustering. DENCLUE, density based spatial clustering, and (c) graph based: hierarchical clustering, chameleon, SNN-DBSCAN.

Furthermore, not only the non-linear models could be enhanced, but also the linear ones. For example, adding interaction terms to a regression model can greatly expand the understanding of the relationships among the variables in the model and allows for more hypotheses to be tested. Interaction effects occur when the effect of one variable depends on the value of another variable, an interaction effect that indicates that a third variable influences the relationship between an independent and dependent variable.

However, the analytical framework from the present research improves the knowledge that is related to heavy metal studies, as well as their efficiency. Thus, it is known that the macro-elements (Ca, Mg, Na, K) are generally determined using the FAAS technique, whereas, for the determination of micro-elements (Zn, Fe, Cu, Ni, Cd, Pb, Cr), the GFAAS is used. From a difficulty point of view, the FAAS are classified as very easy, respectively easy, whereas GFAAS are classified as moderately easy, respectively, difficult. Moreover, the capital and running costs are low for FAAS, whereas, for GFAAS, they are medium to high. Regarding costs per elemental analysis, FAAS scores low costs, while GFAAS registers high costs. Additionally, it is important to mention that sample throughput is 10–15 s per element in the case of FAAS and 3–4 min. per element in the case of GFAAS.

Therefore, this confirms that the analytical framework from the present study, which implies the determination of micro-elements, used as dependent variables, when considering the macro-elements, used as independent variables, improves the efficiency of heavy metal studies.

## 3. Material and Methods

### 3.1. Study Area

**Figure 2:**
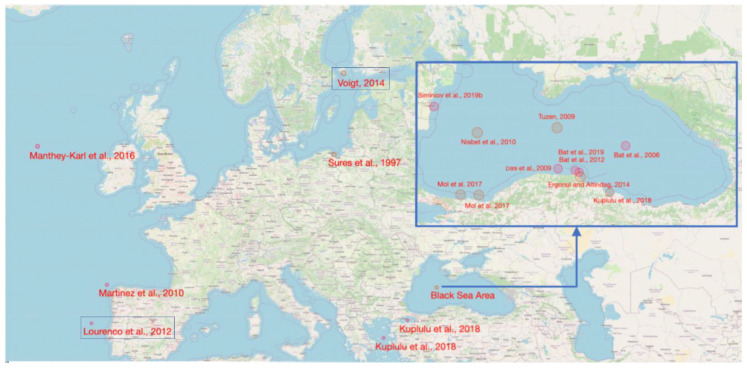
The sampling area of the scientific studies used as dataset for developing the analytical framework (the literature sources which present only data recorded for aquaculture turbot specimens are bordered, while sources that present data for wild turbot specimens are not bordered).

Data that were related to heavy metal concentration in turbot muscle and liver tissues, as reported in Europe (including Turkish Black Sea coast), were collected from the scientific literature [72,73,74,75,76,99,100,101,102,103,104,105,106,107,108]. Figure 2 presents the sampling area reported in the scientific sources used for developing the analytical framework. Strategies for scientific study retrieval and selection were performed according to a specific methodology [104], targeting therefore to obtain a dataset with as many possible samples, while considering especially the studies published in most significant key journals, which have the highest visibility. As limiting factors in the process of scientific paper selection, it can be considered that the extent of searching is determined by the research question and resources available to the research team.

The taxonomic name of the Black Sea turbot has been changed through the 100 years history of its scientific investigation. Numerous names are used concurrently by different groups of researchers [109]. FishBase (www.fishbase.us/summary/Scophthalmusmaeoticus.html) recommends the use of the name *Scophthalmus maeoticus* (Pallas, 1814) for the Black Sea turbot, along with synonym *Psetta maxima maeotica* (Pallas, 1814).

Additional genetic studies are needed in order to specify its position (possibly, on the subspecies level) [109]. Additionally, FishBase (http://fishbase.org/summary/Scophthalmus-maeoticus.html) considers *Scophthalmus maeoticus* (Pallas, 1814) as a synonym of *Scophthalmus maximus* (Linnaeus, 1758). Therefore, all three names that appear in the scientific literature used in present study, *Scophthalmus maeoticus* [106], *Psetta maxima maeotica* [72,75,76,100,101,102,107,109], and *Scophthalmus maximus* [73,74,99,103,108].

### 3.2. Heavy Metal Measurement Methods in Scientific Studies Used to Obtain the Dataset Needed by the Development of the Present Paper Analytical Framework

There are a number of different methods that can be employed to detect metal levels in fish and the other marine animals: Inductively Coupled Plasma Atomic Emission Spectrometric Method (ICP-MS), Flame Atomic Absorption Spectrometric (FAAS), Atomic Absorption Spectrometric with Graphite Furnace (GFAAS), Electro-Thermal Evaporation Inductively Coupled Plasma Mass Spectrometry (ETV-IDICP-MS), Inductively Coupled Plasma Optical Spectrometry (ICP-OES), Inductively Coupled Plasma Spectrometry Having Isotope (ID-ICPMS), and Inductively Coupled Plasma Flame Emission Spectrometry (ICP-AES) [110,111,112,113] The GFAAS is considered to be a suitable method due to its very good detection limit, few spectral interferences and the possibility of automation [114,115,116,117].

Thus, heavy metals in turbot were determined in several scientific studies that were based on FAAS and GFAAS methods [22,72,75,98,102,107,117], followed by ICP-MS [73,74,106,108] and ICP-AES [100], and the collected dataset was used in the development of the present paper analytical framework.

### 3.3. Analytical Framework Methods

Machine learning represents a field of study that is based on a multitude of computational algorithms that are able to define, based on empirical data, formal usable models. As such, through machine learning, a system is giving the ability to acquire and integrate knowledge in order to extend itself by learning new knowledge from the existing one.

Machine learning algorithms are divided in two main classes: supervised and unsupervised algorithms. Supervised learning is performed based on ground truth—prior knowledge of the output values exists in the datasets. Supervised learning aims to define a function that is based on data samples and desired outputs, best approximates the relationship between input and output observable in the data. Unsupervised learning does not have any labeled outputs, with its purpose being to infer the natural structure present within a set of data points. The most common tasks within unsupervised learning are clustering, exploratory analysis for identifying structure in data, and dimensionality reduction, for representing data using less columns or features. In situations where it is impossible to propose trends in the data, unsupervised learning can provide initial insights that can then be used to test individual hypotheses.

Supervised machine learning algorithms are split into two classes—regression algorithms and classification algorithms. As an example, for classification algorithms (predicting object classes) the following can be mentioned: logistic regression, K-Nearest neighbor, support vector machines, naïve Bayes, decision trees, and ensemble learning. As for the regression algorithms used to predict continuous numerical values, the following ones can be mentioned: linear and polynomial regression support vector regression, decision trees, and ensemble learning.

The current research is based on two supervised machine learning algorithms, multiple linear regression and random forest, which were used to both predict heavy metals concentrations in turbot liver and muscle tissues, and to assess the importance of various heavy metals predictors in those predictions.

**Figure 3:**
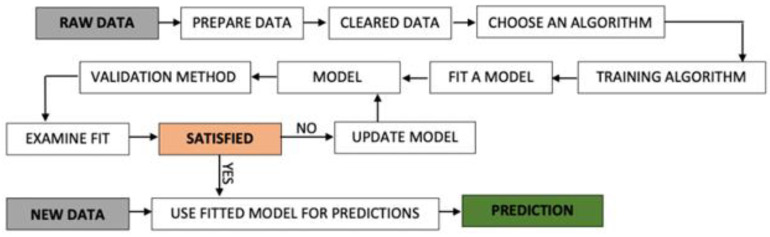
A machine learning typical workflow (original figure).

No matter what machine learning algorithms are used, the workflow that is required for deploying a complete predictive technological solution is the same (Figure 3).

Any machine learning solution starts with raw data, which is data acquired from specific contexts, as it can be observed from Figure 3. In the case it is needed, the data will be prepared (normalized, standardized, missing values imposed) or cleared (e.g., null records deleted). Every algorithm that will be tested will involve a training phase based on a percentage of the dataset (usually 70–80%) that will lead to the development of an initial model that will be validated through performance—goodness of fit metrics. In case the model performs well, it will be used with new data for obtaining new predictions. Otherwise, the model will be updated/fine-tuned until its maximum performance is achieved.

Any machine learning approach displays a certain degree of sensitiveness to environmental changes, or to any new data that are entirely different from what has been used to train the model. Still, the machine learning model creation process is not supposed to be a single time operation. New data should be added to the datasets and the models should be continuously updated. Accordingly, it is not about creating new models, but to retrain the existing ones with data that come from as many scenarios possible as soon as new data are available. Nevertheless, it should not be concluded that the existing models will fail on previously unseen data, it is just that their accuracy could be lower.

Another aspect that should be emphasized is that every machine learning algorithm has different limitations and assumptions. As such, there are models for which the data distribution is important, for example, models that perform optimal when data displays a normal distribution. Still, the current research is not using any of these models so there is no need to graphically display the data distribution. Actually, there are many models for which data distribution is not relevant: Support Vector Machines (SVM), Ensemble models (e.g., Random Forest, Ada Boost), tree-based models, or linear regression (normal, lasso, or ridge). As such, for our current study, it was not mandatory to transform the observed variables through pre-processing techniques in order to obtain normal distribution as linear regression and random forest analysis does not assume normality for either predictors (independent variables) or the outcome (dependent variables). As related to normal distribution, we checked after modelling the residuals of the models if they follow a normal distribution, as this is part of any linear regression model validation.

The current research used supervised machine learning regression algorithms as a methodology that could provide: (a) predicting heavy metals concentration in turbot liver and muscle tissue and (b) assessing the importance of certain heavy metals in predicting another heavy metal turbot tissue concentration. Additionally, it follows the workflow presented in Figure 3, according to these steps: raw data containing heavy metal measurement samples were acquired and stored; raw data were prepared and cleared; random forest regression and multiple linear regression algorithms were chosen to model the data—according to the available number of samples; the models were trained based on 80% of the raw data; models were validated and fine-tuned; models were used to assess predictors importance for different prediction contexts.

For the current research, the data were pre-processed in two ways: (a) NULL values removal—this implied the removal of any dataset record containing NULL values, a record that was supposed to be involved in training a specific model, (b) in the case of multiple linear regression, independent variables were standardized. There are several ways to perform standardization, like subtracting the mean, division by the standard deviation, and the one used in the current research: subtract the mean and then divide by standard deviation. Normally, standardization is mostly required when the regression model contains polynomial or interaction terms. Even if the current research is not using this kind of parameters, standardization was performed, as it helps in answering the question of which of the independent variables have a greater effect on the dependent variable in a multiple regression analysis, when the variables are measured in different units of measurement or the values between the predictors exhibits significant differences. Standardization puts different predictors on the same scale and allows for directly comparing their coefficients. Standardized coefficients represent the mean change in the response given a one standard deviation change in the predictor.

Machine learning algorithms performance is, in most of the cases, related to the volume data that were used in the analysis. The present paper’s approach, which is, using random forest as the main tool for modelling non-linear relations, was chosen because of its ability to handle small datasets, as opposed to other modelling algorithms that are more data greedy (e.g., neural networks, deep learning). When developing a machine learning algorithm, we are mainly interested in two aspects, respectively good precision and good accuracy. In our case, for future enhancement of these two, we would suggest that more data should be collected and more machine learning algorithms should be tested.

For the validation of MLR models, the current research presented three variants of the R square statistical indicator: R-Sq, R-Sq (adj), and R-Sq (pred). The R-sq indicates the goodness of fit, namely how much of the dependent variable variance is explained by the predictors, while the adjusted R-sq penalize the model when many parameters that are not actually contributing to explaining the original variance. The predicted R-squared shows how well a regression model predicts responses for new observations. A key benefit of predicted R-squared is that it can prevent you from overfitting a model. An overfit model contains too many predictors and it starts to model the random noise. Because it is impossible to predict random noise, the predicted R-squared must drop for an overfit model. The current study considered an R-Sq (pred) with a value higher than 60% to be relevant. Besides the predicted R-Sq, *p*-values, and S (standard error of regression) metrics, the multiple linear regressions that are presented in the current research also took the Variance Inflation Factor (VIF) into consideration. VIF helps in measuring the effect of multicollinearity among the predictors. The VIF measures how much the variance of an estimated regression coefficient increases if the predictors are correlated. The reference VIF value was 5, still our models exhibit a VIF parameter far less than 5.

Both multiple linear regression and random forest algorithms are validated while using previously unseen data. Actual values are compared with predicted ones, as can be observed in Figure A1, Figure A2, Figure A3, Figure A4, Figure A5 and Figure A6. When developing a machine learning solution, the overall dataset is split in two: a training dataset and a test dataset. The training dataset, which usually contains 70–80% of the data, is used to identify the ML model, while the test dataset (20–30% of the data) contains ‘fresh’ samples that are used to test and validate the model.

The Random forest machine learning technique that the current research uses provided meaningful models, even when they are trained on small datasets. For random forest algorithm, what matters the most is the number of predictors [118]. For example, in [119], the authors are developing a RF model that is based on 13 predictors.

The models that are presented in the current research requires random concentration data and not time dependent data, as they are not targeting time series analysis. Both linear and non-linear analytical methods were applied in order to identify the existing relations between the parameters used in the present study, as follows.

#### 3.3.1. Multiple Linear Regression Method (MLR)

The linear approach was based on identifying and testing multiple linear regressions models, embedding independent variables that were determined by using stepwise selection methods, having, as the inclusion criteria, the statistical significance at *p* < 0.05. The MLR are excellent for assessing the existing relations between one dependent variable and several independent variables, aiming to fit the data through a linear equation (Equation (1)), as presented below:

(1) $$Y=\alpha_{1}*X_{1}+\alpha_{2}*X_{2}+\ldots +\alpha_{p}*X_{p}+\beta +e$$

where *Y* is the dependent variable; *X*_1_, *X*_2_ … *X_p_*—the *p* independent variables (predictors); *Β*—the intercept indicating the *Y* value when all the predictors are zeros; *α*_1_, *α*_2_ … *α_p_*—the coefficients of predictors, reflecting the contribution of each independent variable in predicting the dependent variable; and, *e*—the residual term indicating the difference between the actual and the fitted response value.

The MLR models offer the possibility to quantify the relationship between the variables, being easy to implement and efficient to train the data. The overfitting is avoided by using dimensionality reduction, regularization, and cross-validation. The optimal features that are to be used by the data modelling process were selected in order for the machine learning scenario models to perform better. Selecting the optimal features is important when the number of features is high, as it is not necessary to use each available feature in implementing the algorithms. Thus, the algorithm was only fed with important features that can explain the dependent variable.

The MLR used in the present paper were developed by using stepwise regression methods in order to reduce model complexity and make it easier to interpret, improve model accuracy by selecting the right predictors subset, and reduce overfitting. Stepwise regression adds or removes individual predictors one at a time based on their statistical significance, building, in this way, the most relevant model. Additionally, the stepwise technique was chosen for its possibility to include the significance level that will be used to accept or not accept a parameter, which is fine-tuning the model.

#### 3.3.2. Non-Linear Models, Based on Random Forest (RF) Algorithm

Because not every parameter can be described through an MLR, in order to determine what the most important parameters are when predicting others (that is assessing the feature importance in a prediction model), in the present study, a tree-based machine learning regression method, namely RF, was applied, instead of polynomial functions. Detailed presentations of RF algorithms can be found in several papers, like [58,120,121,122]. Basically, random forests are ensemble learning algorithms, as described by [123], which use decision trees as base learners. According to Hastie [122], in random forests, the correlation between the trees is reduced and so is the variance of the predictions (i.e., the average of the trees).

An important aspect of our research was to identify/estimate the variables’ importance with respect to the predicted one. As presented by [122,124,125], it is possible to achieve this with RF, through the use of variable importance metrics. As [126] emphasizes, it is possible to rank the predictor variables in terms of relative significance through these metrics. In the present research, feature importance was calculated as the decrease in node impurity weighted by the probability of reaching that node, probability that is calculated by the number of samples that reach the node, divided by the total number of samples. If the value is high, the feature is more important. For each decision tree, the python library calculates node importance (Equation (2)) while using Gini importance, assuming only two child nodes (binary tree):

(2) $$n_{ij}=w_{j}C_{j}-w_{left\left(k\right)}C_{left\left(j\right)}-w_{right\left(j\right)}C_{right\left(j\right)}$$

where $n_{ij}$ represents the importance of node *j*; *w_j_* = weighted number of samples reaching node *j*; *C_j_* = the impurity value of node *j*; *left*(*j*) = child node from left split on node *j*; and *right*(*j*) = child node from right split on node *j*.

The importance for each feature on a decision tree is calculated according to the following formula (Equation (3)):

(3) $$fi_{i}=\frac{\sum_{j:node\;j\;splits\;on\;feature\;i}ni_{j}}{\sum_{k\in all\;nodes}ni_{k}}$$

where $fi_{i}$ is the importance of feature *i*; and $ni_{j}$ = the importance of node *j*.

These values obtained from Equation (3) must be normalized ($normfi_{i}$) to values between 0 and 1 by dividing by the sum of all the feature importance values (Equation (4)).

(4) $$normfi_{i}=\frac{\sum_{j\in all\;trees}normfi_{ij}}{T}$$

The final feature importance, at the RF level, is the average over all the trees. The sum of the feature’s importance value on each tree is calculated and divided by the total number of trees, as in the following equation (Equation (5)):

(5) $$RFfi_{i}=\frac{\sum_{j\in all\;trees}normfi_{ij}}{T}$$

where $RFfi_{i}$ represents the importance of feature *i*, calculated from all trees in the RF model; $normfi_{ij}$ is the normalized feature importance for *i* in tree *j*; and *T* = total number of trees.

In the present research, every RF model was validated while using a mean absolute percentage error (MAPE) based algorithm. The MAPE represents a measure of prediction accuracy of a forecasting method and also a loss function for regression problems in machine learning and it was specifically useful due to its advantages of scale-independency and interpretability, allowing for different models to be directly compared. It expresses the model accuracy according to Equation (6).

(6) $$M=\frac{1}{n}\overset{n}{\underset{t=1}{\sum }}\left|\frac{A_{t}-F_{t}}{A_{t}}\right|$$

where *A_t_* is the actual value; and *F_t_* is the forecast value.

The reason of choosing RF for modelling non-linear parameter relations in the present study were selected and are presented in Table 2.

**Figure A7:**
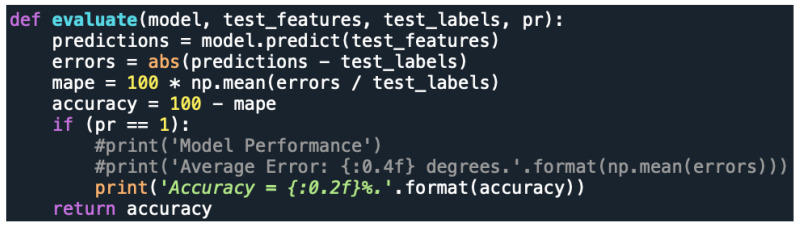
Python code excerpt for implementing random forest evaluation method.

Appendix A presents the Python implementation of the RF evaluation method is emphasized by the code excerpt (Figure A7).

Besides the evaluation procedure, each RF of the model hyperparameters should be fine-tuned in order to obtain the best results. The present research uses the grid-search method in order to achieve this.

For a RF regression model, the following hyperparameters can be adjusted: n_estimators (number of trees in the forest of the model), max_depth (the maximum depth of each tree), min_samples_split (minimum number of samples required to split an internal leaf node), and min_samples_leaf (minimum number of samples required to be at a leaf node).

Therefore, the current research presents, for each relevant random forest model, the following: its precision, the actual model after hyperparameter fine tuning using grid search algorithm, seed selection for maximum accuracy, feature importance according to its weight, and a predicted vs. actual values chart, which shows how the random forest developed model managed to predict previously unseen data as compared with the real data values.

### 3.4. Dataset Descriptive Statistics

The present research is based on a dataset containing a maximum of 22 parameters, describing heavy metal presence in both the turbot muscle and liver tissues. The dataset consists of 63 samples divided into five groups. The first group contains 40 samples from the Black Sea region, which all measured the following variables: As muscle, As liver, Cd muscle, Cd liver, Fe muscle, Fe liver, Cu muscle, Cu liver, Mn muscle, Mn liver, Zn muscle, Zn liver, Ni muscle, Ni liver, Ca muscle, Ca liver, Mg muscle, Mg liver, Na muscle, Na liver, K muscle, K liver, Turbot weight, and Turbot length. The second group contains 44 samples from the Black Sea region, which all measured the following variables: Zn muscle, Cd muscle, Fe muscle, Cu muscle, Ni muscle, and Mn muscle. The third group contains 48 samples from the Black Sea region, that all measured the following variables: Cd muscle, Cu muscle, Zn muscle. The fourth group contains 47 samples from Europe, which all measured the following variables: Cu muscle, Cd muscle, Fe muscle, Mn muscle, Ni muscle, and Zn muscle. The fifth group contains 22 samples from Europe, which all measured the following variables: As muscle, Cd muscle, and Pb muscle. In the last two groups (group 4 and 5), information related to both wild and aquaculture turbot specimens are included, while at the first three groups (group 1, 2, 3) only data reported for wild turbot specimens were used. The decision to include both data from wild and aquaculture turbot can be justified, since, according to scientific literature [99], no significant differences were recorded in terms of heavy metals concentration in turbot tissues, between wild and aquaculture specimens and, also, models that are based on a higher number of samples are more convenient.

Appendix A presents the descriptive statistics for each of the five groups (Table A3, Table A4, Table A5, Table A6 and Table A7).

## 4. Conclusions

The machine learning MLR and non-linear tree-based RF prediction models are identified as being suitable for predicting the heavy metal concentration from both turbot muscle and liver tissues. The models can be used for improving the knowledge and economic efficiency of linked heavy metals food safety and environment pollution studies. The MLR and RF models both complement each other and form a complete heavy metal analytical framework, as MRL evaluates the interactions between the analyzed heavy metals from turbot muscle and liver and RF models manage to accurately predict the required data. The present paper’s analytical framework may be used as a starting point, in order to develop more a complex machine learning analytical framework that involves heavy metals presence in aquatic environment.

The results that were obtained in this paper prove to be useful for obtaining additional information using an already existing dataset. Thus, by applying the prediction models from the present study, it is possible to use a dataset containing the concentrations of macro-elements Na, Mg, Ca, K in turbot muscle, in order to identify, with the least possible error, the concentration of micro-elements. Additionally, the models can be applied for determining the micro-elements when considering other macro and micro-elements and the weight and length of the turbot biological material.

The obtained models can increase the economic efficiency of preliminary studies involving the monitoring of the aquatic environment in order to assess its ecological status and can be used as tool in assessing food safety, including the health risk that is associated with the ingestion of heavy metals following the consumption of fish, since machine learning methods (MLM) are more time and cost efficient, when compared to classical laboratory methods, as mentioned above.

It is recommended to use macro-elements as independent variables for the prediction of micro-elements due to practical and, at the same time, economic reasons, since the analysis of micro-elements involves higher costs and the development of more complex working protocols.

Although the accuracy of MLM is lower when compared to classical laboratory methods, in time, the development of an already existing dataset will generate models with improved prediction precision, a situation that will rise the utility and popularity of these methods among average potential users. More data would certainly help in increasing the precision of the models, while, for increasing the model accuracy, our recommendation would be the fine tuning of various algorithms meta parameters. Special techniques, like the ones used in our research (e.g., grid search, k-fold cross validation), can be used with different models (when sufficient data is available) in order to identify the best parameters for accuracy optimization while avoiding the over-fitting situation, which is the case where the model accurately predicts the training data and poorly performing on unseen data.

When considering the models elaborated in the present research, it can be concluded that all four analyzed macro-nutrients (Ca, K, Mg, and Na) are suitable and have been widely used as independent predictors for determining the micro-nutrients concentration in both turbot muscle and liver tissues. Additionally, among the micro-nutrients, Zn was the most used as an independent predictor, followed by Fe and Ni. However, related to the dependent variable, models for determining the Cd, Cu, Fe, Mn, and Zn micro-elements and K and Mn macro-elements have presented the best results in terms of accuracy of prediction.

Additionally, future studies must aim to integrate new environmental parameters, like water temperature, salinity, or pH value, as well as water and sediments heavy metals concentration, in order to elaborate more complex prediction models, with higher accuracy value.

## Figures and Tables

**Table 1:** Feature importance of elements in all Random Forest (RF) models from current study.

Parameter	Weight 1	Weight 2	Weight 3	Weight 4	Weight 5	Total	Total Per Element
Ca muscle	-	1	2	1	2	6	14
Ca liver	3	2	1	-	2	8	
K muscle	2	2	-	1	1	6	12
K liver	-	2	3	1	-	6	
Zn muscle	1	-	1	1	-	3	13
Zn liver	1	2	4	1	2	10	
Mg muscle	2	-	-	2	1	5	10
Mg liver	1	1	1	-	2	5	
Ni muscle	2	-	-	1	-	3	8
Ni liver		1	2	1	2	6	
Fe muscle	2	2	1	-	1	6	8
Fe liver	-	1	-	1	-	2	
Na muscle	2	-	-	3	-	5	8
Na liver	3	-	-	-	-	3	
Cu muscle	-	1	-	1	1	3	5
Cu liver	-	1	-	-	1	2	
Mn muscle	-	-	-	1	1	2	5
Mn liver	-	-	2	1	-	3	
Cd muscle	-	-	-	1	1	2	5
Cd liver	1	2	-	-	-	3	
As muscle	-	-	2	1	-	3	5
As liver	1	1		--	-	2	

**Table 2:** Reasons for Random Forest use in the present study.

No.	Characteristic	Authors
1	Predictive performance	[58,127]
2	No overfitting	[127]
3	Highly Flexible	[126,128,129]
4	Can capture non-linear dependencies	[126]
5	Robust when noise is present	[127]
6	Formalized predictor significance	[58,127,128]
7	Fast	[128]
8	Suitable for small datasets	[58]
9	Efficient when interactions are present	[126]
10	Small number of model parameters	[58]
11	Stable	[129]
12	Good for high dimensional data	[127]
13	Various type of problems	[58]
14	Straightforward to use	[129]
15	Can handle highly correlated predictor variables	[128]

## Appendix A

**Table A1:** The version of RF models and regressors for the first group MLR models, described in Section 2.2.1. (models 1–8b).

No. of MLR Model	RF Model	RF Regressor
**1**	RF MODEL: Ca muscle–Feature importance: 0.11 for Ca liver, 0.05 for Na liver, 0.02 for Mg liver, 0.01 for Ni liver and 0.01 for K liver; Model Accuracy: 90.27% (MAPE = 9.73%)	RandomForestRegressor(bootstrap = False, ccp_alpha = 0.0, criterion = ‘mse’, max_depth = 40, max_features = 4, max_leaf_nodes = None, max_samples = None, min_impurity_decrease = 0.0, min_impurity_split = None, min_samples_leaf = 2, min_samples_split = 2, min_weight_fraction_leaf = 0.0, n_estimators = 30, n_jobs = None, oob_score = False, random_state = 256, verbose = 0, warm_start = False)
**2**	RF MODEL: Cu liver–Feature importance: 0.06 for Zn liver, 0.04 for Mg liver, 0.03 for Ni liver; Model Accuracy: 95.81% (MAPE = 4.19%)	RandomForestRegressor(bootstrap = True, ccp_alpha = 0.0, criterion = ‘mse’, max_depth = 40, max_features = 4, max_leaf_nodes = None, max_samples = None, min_impurity_decrease = 0.0, min_impurity_split = None, min_samples_leaf = 3, min_samples_split = 2, min_weight_fraction_leaf = 0.0, n_estimators = 110, n_jobs = None, oob_score = False, random_state = 58, verbose = 0, warm_start = False)
**3**	RF MODEL: Fe muscle–Feature importance: 0.06 for Na muscle, 0.05 for K liver, 0.04 for Mn liver, 0.04 for Mg muscle, 0.03 for Ni liver; Model Accuracy: 89.48% (MAPE = 10.52%)	RandomForestRegressor(bootstrap = True, ccp_alpha = 0.0, criterion = ‘mse’, max_depth = 40, max_features = 2, max_leaf_nodes = None, max_samples = None, min_impurity_decrease = 0.0, min_impurity_split = None, min_samples_leaf = 3, min_samples_split = 2, min_weight_fraction_leaf = 0.0, n_estimators = 40, n_jobs = None, oob_score = False, random_state = 80, verbose = 0, warm_start = False)
**4**	RF MODEL: K liver–Feature importance: 0.14 for Na liver, 0.11 for Ca liver, 0.11 for Ca muscle, 0.09 for Fe liver, 0.04 for Mg liver; Model Accuracy: 97.66% (MAPE = 2.34%)	RandomForestRegressor(bootstrap = False, ccp_alpha = 0.0, criterion = ‘mse’, max_depth = 40, max_features = 3, max_leaf_nodes = None, max_samples = None, min_impurity_decrease = 0.0, min_impurity_split = None, min_samples_leaf = 2, min_samples_split = 2, min_weight_fraction_leaf = 0.0, n_estimators = 30, n_jobs = None, oob_score = False, random_state = 124, verbose = 0, warm_start = False)
**5**	RF MODEL: Mg muscle–Feature importance: 0.13 for Na muscle, 0.08 for Zn liver, 0.03 for Ni liver, 0.02 for Cu muscle, 0.02 for Fe muscle; Model Accuracy: 97.01% (MAPE = 2.99%)	RandomForestRegressor(bootstrap = True, ccp_alpha = 0.0, criterion = ‘mse’, max_depth = 40, max_features = 4, max_leaf_nodes = None, max_samples = None, min_impurity_decrease = 0.0, min_impurity_split = None, min_samples_leaf = 3, min_samples_split = 2, min_weight_fraction_leaf = 0.0, n_estimators = 60, n_jobs = None, oob_score = False, random_state = 297, verbose = 0, warm_start = False)
**6**	RF MODEL: Na liver–Feature importance: 0.16 for Ca liver, 0.09 for Fe muscle, 0.08 for Mg liver, 0.07 for K liver, 0.02 for Mg muscle; Model Accuracy: 98.48% (MAPE = 1.52%)	RandomForestRegressor(bootstrap = True, ccp_alpha = 0.0, criterion = ‘mse’, max_depth = 40, max_features = 2, max_leaf_nodes = None, max_samples = None, min_impurity_decrease = 0.0, min_impurity_split = None, min_samples_leaf = 2, min_samples_split = 2, min_weight_fraction_leaf = 0.0, n_estimators = 30, n_jobs = None, oob_score = False, random_state = 115, verbose = 0, warm_start = False)
**7**	RF MODEL: Na muscle–Feature importance: 0.08 for Fe muscle, 0.06 for Zn liver, 0.04 for Ca muscle, 0.03 for Mg muscle, 0.03 for Ca liver; Model Accuracy: 97.09% (MAPE = 2.91%)	RandomForestRegressor(bootstrap = True, ccp_alpha = 0.0, criterion = ‘mse’, max_depth = 40, max_features = 4, max_leaf_nodes = None, max_samples = None, min_impurity_decrease = 0.0, min_impurity_split = None, min_samples_leaf = 2, min_samples_split = 2, min_weight_fraction_leaf = 0.0, n_estimators = 50, n_jobs = None, oob_score = False, random_state = 273, verbose = 0, warm_start = False)
**8a,b**	RF MODEL: Zn liver–Feature importance: 0.04 for Ca liver, 0.01 for Cd liver, 0.01 for Zn muscle, 0.01 for Mn muscle; Model Accuracy: 97.78% (MAPE = 2.22%)	RandomForestRegressor(bootstrap = True, ccp_alpha = 0.0, criterion = ‘mse’, max_depth = 40, max_features = 4, max_leaf_nodes = None, max_samples = None, min_impurity_decrease = 0.0, min_impurity_split = None, min_samples_leaf = 2, min_samples_split = 2, min_weight_fraction_leaf = 0.0, n_estimators = 40, n_jobs = None, oob_score = False, random_state = 299, verbose = 0, warm_start = False)

**Table A2:** The RF regressors.

Model No.	RF Model Regressor
**9**	RandomForestRegressor(bootstrap = True, ccp_alpha = 0.0, criterion = ‘mse’, max_depth = 100, max_features = 4, max_leaf_nodes = None, max_samples = None, min_impurity_decrease = 0.0, min_impurity_split = None, min_samples_leaf = 1, min_samples_split = 3, min_weight_fraction_leaf = 0.0, n_estimators = 200, n_jobs = None, oob_score = False, random_state = 116, verbose = 0, warm_start = False)
**10**	RandomForestRegressor(bootstrap = True, ccp_alpha = 0.0, criterion = ‘mse’, max_depth = 100, max_features = 4, max_leaf_nodes = None, max_samples = None, min_impurity_decrease = 0.0, min_impurity_split = None, min_samples_leaf = 3, min_samples_split = 2, min_weight_fraction_leaf = 0.0, n_estimators = 200, n_jobs = None, oob_score = False, random_state = 278, verbose = 0, warm_start = False)
**11**	RandomForestRegressor(bootstrap = True, ccp_alpha = 0.0, criterion = ‘mse’, max_depth = 100, max_features = 4, max_leaf_nodes = None, max_samples = None, min_impurity_decrease = 0.0, min_impurity_split = None, min_samples_leaf = 3, min_samples_split = 2, min_weight_fraction_leaf = 0.0, n_estimators = 200, n_jobs = None, oob_score = False, random_state = 15, verbose = 0, warm_start = False)
**12**	RandomForestRegressor(bootstrap = True, ccp_alpha = 0.0, criterion = ‘mse’, max_depth = 100, max_features = 2, max_leaf_nodes = None, max_samples = None, min_impurity_decrease = 0.0, min_impurity_split = None, min_samples_leaf = 1, min_samples_split = 2, min_weight_fraction_leaf = 0.0, n_estimators = 100, n_jobs = None, oob_score = False, random_state = 237, verbose = 0, warm_start = False)
**13**	RandomForestRegressor(bootstrap = True, ccp_alpha = 0.0, criterion = ‘mse’, max_depth = 40, max_features = 2, max_leaf_nodes = None, max_samples = None, min_impurity_decrease = 0.0, min_impurity_split = None, min_samples_leaf = 1, min_samples_split = 2, min_weight_fraction_leaf = 0.0, n_estimators = 50, n_jobs = None, oob_score = False, random_state = 227, verbose = 0, warm_start = False)
**14**	RandomForestRegressor(bootstrap = True, ccp_alpha = 0.0, criterion = ‘mse’, max_depth = 40, max_features = 2, max_leaf_nodes = None, max_samples = None, min_impurity_decrease = 0.0, min_impurity_split = None, min_samples_leaf = 1, min_samples_split = 2, min_weight_fraction_leaf = 0.0, n_estimators = 90, n_jobs = None, oob_score = False, random_state = 214, verbose = 0, warm_start = False)
**15**	RandomForestRegressor(bootstrap = True, ccp_alpha = 0.0, criterion = ‘mse’, max_depth = 100, max_features = 3, max_leaf_nodes = None, max_samples = None, min_impurity_decrease = 0.0, min_impurity_split = None, min_samples_leaf = 1, min_samples_split = 3, min_weight_fraction_leaf = 0.0, n_estimators = 100, n_jobs = None, oob_score = False, random_state = 43, verbose = 0, warm_start = False)
**16**	RandomForestRegressor(bootstrap = True, ccp_alpha = 0.0, criterion = ‘mse’, max_depth = 100, max_features = 2, max_leaf_nodes = None, max_samples = None, min_impurity_decrease = 0.0, min_impurity_split = None, min_samples_leaf = 1, min_samples_split = 2, min_weight_fraction_leaf = 0.0, n_estimators = 100, n_jobs = None, oob_score = False, random_state = 201, verbose = 0, warm_start = False)
**17**	RandomForestRegressor(bootstrap = True, ccp_alpha = 0.0, criterion = ‘mse’, max_depth = 100, max_features = 4, max_leaf_nodes = None, max_samples = None, min_impurity_decrease = 0.0, min_impurity_split = None, min_samples_leaf = 2, min_samples_split = 2, min_weight_fraction_leaf = 0.0, n_estimators = 100, n_jobs = None, oob_score = False, random_state = 93, verbose = 0, warm_start = False)
**18**	RandomForestRegressor(bootstrap = True, ccp_alpha = 0.0, criterion = ‘mse’, max_depth = 100, max_features = 4, max_leaf_nodes = None, max_samples = None, min_impurity_decrease = 0.0, min_impurity_split = None, min_samples_leaf = 2, min_samples_split = 2, min_weight_fraction_leaf = 0.0, n_estimators = 100, n_jobs = None, oob_score = False, random_state = 223, verbose = 0, warm_start = False)
**19**	RandomForestRegressor(bootstrap = True, ccp_alpha = 0.0, criterion = ‘mse’, max_depth = 100, max_features = 3, max_leaf_nodes = None, max_samples = None, min_impurity_decrease = 0.0, min_impurity_split = None, min_samples_leaf = 2, min_samples_split = 2, min_weight_fraction_leaf = 0.0, n_estimators = 100, n_jobs = None, oob_score = False, random_state = 192, verbose = 0, warm_start = False)
**20**	RandomForestRegressor(bootstrap = True, ccp_alpha = 0.0, criterion = ‘mse’, max_depth = 100, max_features = 2, max_leaf_nodes = None, max_samples = None, min_impurity_decrease = 0.0, min_impurity_split = None, min_samples_leaf = 3, min_samples_split = 2, min_weight_fraction_leaf = 0.0, n_estimators = 200, n_jobs = None, oob_score = False, random_state = 206, verbose = 0, warm_start = False)
**21**	RandomForestRegressor(bootstrap = True, ccp_alpha = 0.0, criterion = ‘mse’, max_depth = None, max_features = ‘auto’, max_leaf_nodes = None, max_samples = None, min_impurity_decrease = 0.0, min_impurity_split = None, min_samples_leaf = 1, in_samples_split = 2, min_weight_fraction_leaf = 0.0, n_estimators = 100, n_jobs = None, oob_score = False, random_state = 173, verbose = 0, warm_start = False)
**22**	RandomForestRegressor(bootstrap = True, ccp_alpha = 0.0, criterion = ‘mse’, max_depth = 40, max_features = 4, max_leaf_nodes = None, max_samples = None, min_impurity_decrease = 0.0, min_impurity_split = None, min_samples_leaf = 1, min_samples_split = 2, min_weight_fraction_leaf = 0.0, n_estimators = 70, n_jobs = None, oob_score = False, random_state = 171, verbose = 0, warm_start = False)
**23**	RandomForestRegressor(bootstrap = True, ccp_alpha = 0.0, criterion = ‘mse’, max_depth = 40, max_features = 3, max_leaf_nodes = None, max_samples = None, min_impurity_decrease = 0.0, min_impurity_split = None, min_samples_leaf = 2, min_samples_split = 2, min_weight_fraction_leaf = 0.0, n_estimators = 100, n_jobs = None, oob_score = False, random_state = 29, verbose = 0, warm_start = False)
**24**	RandomForestRegressor(bootstrap = False, ccp_alpha = 0.0, criterion = ‘mse’, max_depth = 40, max_features = 3, max_leaf_nodes = None, max_samples = None, min_impurity_decrease = 0.0, min_impurity_split = None, min_samples_leaf = 2, min_samples_split = 2, min_weight_fraction_leaf = 0.0, n_estimators = 30, n_jobs = None, oob_score = False, random_state = 237, verbose = 0, warm_start = False)
**25**	RandomForestRegressor(bootstrap = True, ccp_alpha = 0.0, criterion = ‘mse’, max_depth = None, max_features = ‘auto’, max_leaf_nodes = None, max_samples = None, min_impurity_decrease = 0.0, min_impurity_split = None, min_samples_leaf = 1, min_samples_split = 2, min_weight_fraction_leaf = 0.0, n_estimators = 100, n_jobs = None, oob_score = False, random_state = 227, verbose = 0, warm_start = False)
**26**	RandomForestRegressor(bootstrap = True, ccp_alpha = 0.0, criterion = ‘mse’, max_depth = None, max_features = ‘auto’, max_leaf_nodes = None, max_samples = None, min_impurity_decrease = 0.0, min_impurity_split = None, min_samples_leaf = 1, min_samples_split = 2, min_weight_fraction_leaf = 0.0, n_estimators = 100, n_jobs = None, oob_score = False, random_state = 143, verbose = 0, warm_start = False)
**27**	RandomForestRegressor(bootstrap = True, ccp_alpha = 0.0, criterion = ‘mse’, max_depth = None, max_features = ‘auto’, max_leaf_nodes = None, max_samples = None, min_impurity_decrease = 0.0, min_impurity_split = None, min_samples_leaf = 1, min_samples_split = 2, min_weight_fraction_leaf = 0.0, n_estimators = 100, n_jobs = None, oob_score = False, random_state = 45, verbose = 0, warm_start = False)
**28**	RandomForestRegressor(bootstrap = True, ccp_alpha = 0.0, criterion = ‘mse’, max_depth = 40, max_features = 3, max_leaf_nodes = None, max_samples = None, min_impurity_decrease = 0.0, min_impurity_split = None, min_samples_leaf = 3, min_samples_split = 2, min_weight_fraction_leaf = 0.0, n_estimators = 30, n_jobs = None, oob_score = False, random_state = 104, verbose = 0, warm_start = False)
**29**	RandomForestRegressor(bootstrap = True, ccp_alpha = 0.0, criterion = ‘mse’, max_depth = 40, max_features = 3, max_leaf_nodes = None, max_samples = None, min_impurity_decrease = 0.0, min_impurity_split = None, min_samples_leaf = 2, min_samples_split = 2, min_weight_fraction_leaf = 0.0, n_estimators = 110, n_jobs = None, oob_score = False, random_state = 223, verbose = 0, warm_start = False)
**30**	RandomForestRegressor(bootstrap = True, ccp_alpha = 0.0, criterion = ‘mse’, max_depth = None, max_features = ‘auto’, max_leaf_nodes = None, max_samples = None, min_impurity_decrease = 0.0, min_impurity_split = None, min_samples_leaf = 1, min_samples_split = 2, min_weight_fraction_leaf = 0.0, n_estimators = 100, n_jobs = None, oob_score = False, random_state = 76, verbose = 0, warm_start = False)
**31**	RandomForestRegressor(bootstrap = True, ccp_alpha = 0.0, criterion = ‘mse’, max_depth = 40, max_features = 2, max_leaf_nodes = None, max_samples = None, min_impurity_decrease = 0.0, min_impurity_split = None, min_samples_leaf = 3, min_samples_split = 2, min_weight_fraction_leaf = 0.0, n_estimators = 60, n_jobs = None, oob_score = False, random_state = 192, verbose = 0, warm_start = False)
**32**	RandomForestRegressor(bootstrap = True, ccp_alpha = 0.0, criterion = ‘mse’, max_depth = 40, max_features = 4, max_leaf_nodes = None, max_samples = None, min_impurity_decrease = 0.0, min_impurity_split = None, min_samples_leaf = 3, min_samples_split = 2, min_weight_fraction_leaf = 0.0, n_estimators = 50, n_jobs = None, oob_score = False, random_state = 273, verbose = 0, warm_start = False)
**33**	RandomForestRegressor(bootstrap = True, ccp_alpha = 0.0, criterion = ‘mse’, max_depth = 100, max_features = 2, max_leaf_nodes = None, max_samples = None, min_impurity_decrease = 0.0, min_impurity_split = None, min_samples_leaf = 1, min_samples_split = 2, min_weight_fraction_leaf = 0.0, n_estimators = 100, n_jobs = None, oob_score = False, random_state = 93, verbose = 0, warm_start = False)
**34**	RandomForestRegressor(bootstrap = True, ccp_alpha = 0.0, criterion = ‘mse’, max_depth = 100, max_features = 4, max_leaf_nodes = None, max_samples = None, min_impurity_decrease = 0.0, min_impurity_split = None, min_samples_leaf = 1, min_samples_split = 3, min_weight_fraction_leaf = 0.0, n_estimators = 90, n_jobs = None, oob_score = False, random_state = 11, verbose = 0, warm_start = False)
**35**	RandomForestRegressor(bootstrap = True, ccp_alpha = 0.0, criterion = ‘mse’, max_depth = 80, max_features = 2, max_leaf_nodes = None, max_samples = None, min_impurity_decrease = 0.0, min_impurity_split = None, min_samples_leaf = 1, min_samples_split = 3, min_weight_fraction_leaf = 0.0, n_estimators = 90, n_jobs = None, oob_score = False, random_state = 74, verbose = 0, warm_start = False)
**36**	RandomForestRegressor(bootstrap = True, ccp_alpha = 0.0, criterion = ‘mse’, max_depth = 50, max_features = 2, max_leaf_nodes = None, max_samples = None, min_impurity_decrease = 0.0, min_impurity_split = None, min_samples_leaf = 3, min_samples_split = 2, min_weight_fraction_leaf = 0.0, n_estimators = 90, n_jobs = None, oob_score = False, random_state = 54, verbose = 0, warm_start = False)
**37**	RandomForestRegressor(bootstrap = True, ccp_alpha = 0.0, criterion = ‘mse’, max_depth = None, max_features = ‘auto’, max_leaf_nodes = None, max_samples = None, min_impurity_decrease = 0.0, min_impurity_split = None, min_samples_leaf = 1, min_samples_split = 2, min_weight_fraction_leaf = 0.0, n_estimators = 100, n_jobs = None, oob_score = False, random_state = 102, verbose = 0, warm_start = False)
**39**	RandomForestRegressor(bootstrap = True, ccp_alpha = 0.0, criterion = ‘mse’, max_depth = 40, max_features = 2, max_leaf_nodes = None, max_samples = None, min_impurity_decrease = 0.0, min_impurity_split = None, min_samples_leaf = 1, min_samples_split = 2, min_weight_fraction_leaf = 0.0, n_estimators = 80, n_jobs = None, oob_score = False, random_state = 17, verbose = 0, warm_start = False)
**40**	RandomForestRegressor(bootstrap = True, ccp_alpha = 0.0, criterion = ‘mse’, max_depth = 40, max_features = 3, max_leaf_nodes = None, max_samples = None, min_impurity_decrease = 0.0, min_impurity_split = None, min_samples_leaf = 3, min_samples_split = 2, min_weight_fraction_leaf = 0.0, n_estimators = 40, n_jobs = None, oob_score = False, random_state = 284, verbose = 0, warm_start = False)
**41**	RandomForestRegressor(bootstrap = False, ccp_alpha = 0.0, criterion = ‘mse’, max_depth = 40, max_features = 2, max_leaf_nodes = None, max_samples = None, min_impurity_decrease = 0.0, min_impurity_split = None, min_samples_leaf = 1, min_samples_split = 2, min_weight_fraction_leaf = 0.0, n_estimators = 30, n_jobs = None, oob_score = False, random_state = 54, verbose = 0, warm_start = False)
**42**	RandomForestRegressor(bootstrap = True, ccp_alpha = 0.0, criterion = ‘mse’, max_depth = 40, max_features = 2, max_leaf_nodes = None, max_samples = None, min_impurity_decrease = 0.0, min_impurity_split = None, min_samples_leaf = 1, min_samples_split = 2, min_weight_fraction_leaf = 0.0, n_estimators = 100, n_jobs = None, oob_score = False, random_state = 63, verbose = 0, warm_start = False)
**43**	RandomForestRegressor(bootstrap = False, ccp_alpha = 0.0, criterion = ‘mse’, max_depth = 40, max_features = 3, max_leaf_nodes = None, max_samples = None, min_impurity_decrease = 0.0, min_impurity_split = None, min_samples_leaf = 1, min_samples_split = 2, min_weight_fraction_leaf = 0.0, n_estimators = 30, n_jobs = None, oob_score = False, random_state = 22, verbose = 0, warm_start = False)

**Table A3:** Descriptive statistics of first group dataset.

Variable	Unit	Mean	SE Mean	StDev	Min.	Q1	Median	Q3	Max.
As muscle	µg g^−1^ Fresh weight (F.W.)	3.82	0.18	1.14	2.15	2.79	3.69	4.66	6.32
Cd muscle	µg g^−1^ F.W.	0.03	0.00	0.00	0.03	0.03	0.03	0.03	0.04
Fe muscle	µg g^−1^ F.W.	9.13	0.58	3.68	4.33	6.26	8.40	12.84	15.87
Cu muscle	µg g^−1^ F.W.	0.16	0.00	0.01	0.14	0.14	0.16	0.17	0.18
Mn muscle	µg g^−1^ F.W.	0.17	0.01	0.06	0.04	0.15	0.19	0.21	0.27
Zn muscle	µg g^−1^ F.W.	12.18	0.47	2.99	6.17	10.20	13.14	14.39	16.13
Ni muscle	µg g^−1^ F.W.	0.11	0.00	0.03	0.05	0.09	0.11	0.13	0.17
Ca muscle	µg g^−1^ F.W.	176.84	19.63	124.14	52.49	79.52	100.94	287.07	435.90
Mg muscle	µg g^−1^ F.W.	518.08	7.47	47.25	438.42	479.67	517.38	551.31	608.88
Na muscle	µg g^−1^ F.W.	1116.55	31.99	202.32	831.94	917.89	1123.59	1319.56	1394.43
K muscle	µg g^−1^ F.W.	6001.23	38.72	244.91	5640.17	5778.49	5998.94	6191.85	6453.03
As liver	µg g^−1^ F.W.	8.42	0.62	3.92	3.91	4.78	7.44	10.67	17.65
Cd liver	µg g^−1^ F.W.	0.10	0.00	0.02	0.05	0.08	0.09	0.12	0.13
Fe liver	µg g^−1^ F.W.	60.34	1.69	10.68	42.12	52.39	60.16	69.88	79.81
Cu liver	µg g^−1^ F.W.	3.10	0.07	0.42	2.51	2.77	3.00	3.42	3.89
Mn liver	µg g^−1^ F.W.	0.62	0.04	0.27	0.02	0.44	0.61	0.80	1.10
Zn liver	µg g^−1^ F.W.	28.63	0.40	2.50	25.27	26.45	28.01	30.74	33.93
Ni liver	µg g^−1^ F.W.	0.17	0.00	0.03	0.13	0.14	0.17	0.20	0.21
Ca liver	µg g^−1^ F.W.	85.89	4.56	28.82	51.64	58.76	82.24	115.01	121.73
Mg liver	µg g^−1^ F.W.	434.82	14.94	94.52	334.09	344.36	406.76	535.68	599.18
Na liver	µg g^−1^ F.W.	1511.55	23.77	150.32	1217.21	1419.67	1548.77	1645.93	1672.52
K liver	µg g^−1^ F.W.	4889.04	118.62	750.23	3281.69	4252.79	5204.22	5533.91	5580.09
Turbot Weight	kg	1.39	0.02	0.15	1.20	1.26	1.36	1.48	1.70
Turbot Length	cm	43.46	0.31	1.94	40.20	41.77	43.70	44.98	46.80

**Table A4:** Descriptive statistics of second group dataset.

Variable (µg g^−1^ F.W.)	Mean	SE Mean	StDev	Min.	Q1	Median	Q3	Max.
Cd muscle	0.04	0.00	0.01	0.02	0.03	0.03	0.03	0.10
Fe muscle	11.57	1.23	8.23	4.33	6.56	9.33	13.62	39.84
Cu muscle	0.38	0.12	0.83	0.14	0.15	0.16	0.17	5.05
Mn muscle	1.00	0.55	3.67	0.04	0.17	0.20	0.21	24.22
Zn muscle	14.67	1.23	8.26	6.17	10.81	13.49	14.99	45.20
Ni muscle	0.50	0.17	1.14	0.05	0.09	0.12	0.14	4.50

**Table A5:** Descriptive statistics of third group dataset.

Variable (µg g^−1^ F.W.)	Mean	SE Mean	StDev	Min.	Q1	Median	Q3	Max.
Cd muscle	0.03	0.00	0.01	0.01	0.03	0.03	0.03	0.10
Cu muscle	0.55	0.16	1.11	0.14	0.15	0.16	0.17	5.18
Zn muscle	15.31	1.20	8.40	6.17	11.30	13.63	15.43	45.20

**Table A6:** Descriptive statistics of fourth group dataset.

Variable (µg g^−1^ F.W.)	Mean	SE Mean	StDev	Min.	Q1	Median	Q3	Max.
Cd muscle	0.03	0.00	0.01	0.01	0.03	0.03	0.03	0.10
Fe muscle	10.67	1.09	7.42	2.60	6.09	9.17	13.46	39.84
Cu muscle	0.37	0.12	0.82	0.14	0.15	0.16	0.17	5.05
Mn muscle	0.93	0.53	3.61	0.04	0.17	0.20	0.22	24.22
Zn muscle	13.91	1.01	6.86	6.17	10.41	13.42	14.98	45.20
Ni muscle	0.41	0.15	1.03	0.02	0.08	0.11	0.14	4.50

**Table A7:** Descriptive statistics of fifth group dataset.

Variable (µg g^−1^ F.W.)	Mean	SE Mean	StDev	Min.	Q1	Median	Q3	Max.
Pb muscle	0.24	0.08	0.25	0.03	0.10	0.17	0.28	0.85
Cd muscle	0.05	0.01	0.04	0.01	0.01	0.03	0.10	0.11
As muscle	0.91	0.27	0.81	0.15	0.30	0.61	1.58	2.53
